# India Policy Insights: Estimates of Population Health and Social Determinants Indicators Across Policy Units

**DOI:** 10.1038/s41597-025-05923-8

**Published:** 2025-09-30

**Authors:** Sunil Rajpal, Soohyeon Ko, George Leckie, Devika Jain, Jeffrey C. Blossom, Rockli Kim, S. V. Subramanian

**Affiliations:** 1 Department of Economics, FLAME University, Pune, India; 2 Department of Social and Behavioral Sciences, Harvard T. H. Chan School of Public Health, Boston, MA USA; 3 Centre for Multilevel Modelling, University of Bristol, Bristol, UK; 4 Centre for Geographic Analysis, Harvard University, Cambridge, MA USA; 5 Division of Health Policy and Management, College of Health Science, Korea University, Seoul, South Korea; 6 Interdisciplinary Program in Precision Public Health, Department of Public Health Sciences, Graduate School of Korea University, Seoul, South Korea; 7 Harvard Center for Population and Development Studies, Cambridge, MA 02138 USA

**Keywords:** Developing world, Interdisciplinary studies

## Abstract

Achieving global development targets will largely depend on improvements made by developing and populous countries like India. Given the huge population base and diversely wide landscape of India, stakeholders and policy-makers at smaller geographic levels must be equipped with data to understand their respective territories and the target sub-groups. This paper presents the output of *India Policy Insights*; a project aimed at offering a comprehensive dataset of 122 key indicators on population health and social determinants across 720 districts and 543 Parliamentary Constituencies (PCs) in India. Drawing upon the household survey data from two rounds (2016 and 2021) of National Family Health Surveys along with other government-backed repositories, we provide district- and PC-specific prevalence estimates (%), progress in the prevalence (percentage points) for 122 indicators, and indicator-specific headcount numbers (for 2021). These data enable the tracking of developmental progress in India and, in doing so, will facilitate informed policy discussions and promote public engagement.

## Background & Summary

Advancements in social science have gained substantial momentum in utilizing big data, increasingly contributing to evidence-based policymaking^1,2^. The demand for data-based empirical insights in public policymaking has increased tremendously at all levels in the last few decades. For instance, the first set of global targets for achieving critical goals concerning population health and well-being commenced with the establishment of the Millennium Development Goals (MDGs) in 2000, which were targeted to be achieved by 2015^3^. These MDGs further paved the way for the formulation of the Sustainable Development Goals (SDGs) in 2015, to measure and foster economic development, social welfare, and environmental sustainability, with specific goals to be accomplished by 2030^4^.

Such international commitments toward achieving commonly accepted metric-based goals are targeted, monitored, and assessed at the country level. However, national-level achievement of such targets is contingent on the progress made at smaller geographic and administrative units within the country. Importantly, most of the development programs are implemented at the local level targeting smaller geographic areas^5^. It is therefore critical for policy stakeholders (bureaucrats and politicians) to consider smaller geographic units for assessing the progress of their jurisdiction to these national and international targets.

Global progress towards development and well-being will largely depend on improvements made by developing and populous countries, like India^6^. India with its huge population base and a young demographic profile can largely drive the global development metrics. However, the availability of adequate datasets on population health and social determinants in India remains limited, especially for smaller administrative geographies^7,8^. While there may be several reasons for this, India’s vast geography and huge population are safe to assume as notable constraints for such low data frequency. Consequently, most development programs (directly or indirectly linked to SDGs) in India are designed based on sample survey datasets, like the National Family Health Survey (NFHS) – equivalent to Demographic and Health Surveys (DHS) for India^5^. While policy utilization of these datasets is primarily conducted at higher geographical levels (national or state), insights on progress are warranted at more granular geographic levels, specifically districts and Parliamentary Constituencies (PCs).

In India, administrative geography is structured in a hierarchical and nested manner, enabling effective governance across its vast and diverse territory. At the topmost level is the Union Government, overseeing the entire country. Below it, India is divided into States and Union Territories (UTs), which serve as the primary administrative divisions. Each state or UT is further subdivided into districts, which are key units for governance, and development planning. Within districts, there are sub-districts or tehsils/talukas, followed by blocks, and finally villages in rural areas and wards in urban areas. This nested structure ensures a cascading model of governance, where responsibilities and resources flow from the central government down to the grassroots, facilitating localized administration and the implementation of policies tailored to regional needs. Given this, districts are the primary point of contact between the administration and the population, where policies are closely tailored to address local needs.

Along similar lines, PCs in India are geographical electoral constituencies from which representatives are elected to the Lok Sabha, the lower house of India’s bicameral Parliament. They hold substantial importance as they are the core units of electoral democracy in India, and the elected Members of Parliament (MPs) represent the voice, interests, and development needs of their constituents at the national level. As PCs often span multiple districts, such data is essential to bridge gaps and enable more precise advocacy and resource allocation. For example, effective utilization of the funds from the ‘Member of Parliament Local Area Development Scheme’ (MPLADS). Further, PC-level estimates can offer insights into disparity across larger areas at the constituency level. For example, while significant national-level progress has been made in areas of sanitation and neonatal mortality, approximately 119 and 218 PCs, respectively, remain off the SDG target in these areas^9^. The key difference between districts and PCs in India is that districts serve the purpose of administrative management and service delivery (by District Collector / District Magistrate, and other bureaucrats), while PCs serve the political representation by the Elected Member of Parliament.

India’s struggle persists in addressing population health and social determinants at local levels such as districts and PCs. To effectively govern and serve these populations, statistically reliable data and evidence-based policies are essential. National and regional datasets about various indicators of population health and social determinants are available for the Indian context, such as the NFHS^10^, National Sample Survey^11^, Indian Health and Development Survey^12^, Longitudinal Ageing Study India^13^, Census of India^14^, and more. However, there remains a pressing need for a comprehensive dataset enabling academics, policymakers as well as the general population to synthesize, analyze, and visualize the data in a geographically relevant and policy-driven context.

The Indian Policy Insights (IPI) project aimed to address this need by offering a comprehensive dataset of 122 key indicators on population health and social determinants across 720 districts and 543 PCs in India. Given the non-availability of district-level and constituency-level data on key indicators of population health and social determinants in India, the IPI dataset is the first systematic attempt to bridge this gap by providing indirect estimates from sample survey data. Further, the project contributes towards providing the datasets for the most updated number of districts (adjusting for change in the geographical boundaries over time). The key motivation of the IPI initiative is to empower academics and policymakers mainly at the district and PC levels to provide user-friendly, evidence-based data and tools to assess and address local population health and development challenges. The initiative promotes public engagement by offering resources that facilitate informed policy discussions and enable the tracking of developmental progress. IPI makes its resources fully accessible to the public to encourage their informed use. The project was envisaged to serve as a comprehensive data platform leveraging novel statistical techniques to generate geo-visualized data for 122 indicators of population health and social determinants. The platform integrates data from prominent sources like the NFHS, Census projections, and shapefiles from Bharatmaps, among others, to ensure that users can effectively explore population health, comparisons and policy performance metrics.

The following sections document the methodology and potential usage of IPI datasets to understand the burden and geographic variation in a variety of outcomes from child health to nutrition to social infrastructure. It provides an example of how our dataset was created and can be utilized to investigate key development indicators at small-area levels in India.

## Methods

### Data sources

The process for estimating the 122 indicators across seven key domains – healthcare, maternal health and family planning, morbidity, and mortality, clinical and anthropometric aspects of nutrition, dietary nutrition, social infrastructure, and socio-economic profile – relied on several data sources, but primarily the NFHS datasets. Other data sources were used to estimate headcount and update the geographic boundaries for districts and PCs. This classification enabled a comprehensive exploration of various health and population factors across districts and PCs. The full list of indicators, along with their definitions are detailed in Supplementary Table 1.

#### National family health surveys

The household and individual-level information on key indicators was sourced from two rounds of the NFHS — NFHS-4 (2015–16; hereafter, NFHS-2016) and NFHS-5 (2019–21; hereafter, NFHS-2021)^15,16^. The NFHS-2016 survey was conducted between January 20, 2015, and December 4, 2016, while the NFHS-2021 survey was carried out in two phases: Phase I from June 17, 2019, to January 30, 2020, and Phase II from January 2, 2020, to April 30, 2021. These were the most recent survey rounds, making them relevant from a policy perspective at the local level. The NFHS is part of the global DHS initiative, which has conducted over 400 surveys across 90 + countries, offering vital data on health, population, and nutrition indicators.

In India, the NFHS sample represents the population using a multistage, stratified sampling design, providing data on households and individuals living within all of India’s districts and states/UTs. The NFHS-2016 and NFHS-2021 surveys used a two-stage probabilistic sampling approach, first selecting Primary Sampling Units (PSUs) such as villages or urban census blocks using probability proportional to size, and then randomly selecting households within PSUs using systematic sampling. NFHS-5 included 636,699 households, compared to 601,509 in NFHS-2016, and maintained the same sampling methodology across India, although some conflict-affected areas were excluded. Various NFHS files (module recodes) were utilized based on specific indicators of interest. Household indicators were extracted from the Population Recode (PR), child-related data from the Children’s Recode (KR), women-related data from the Individual Recode (IR), and men-related data from the Men’s Recode (MR).

#### Census of India

To compute the indicator-specific headcounts (only for NFHS-2021), we used 2011–2036 population projections from the Census of India^17^. Initiated since 1958, these projections are provided by the Office of the Registrar General and Census Commissioner of India using the cohort component method, a widely recognized approach that incorporates fertility, mortality, and migration rates. The 2011–2036 projections are based on data from the 2011 Census and the Sample Registration System, which provides data on fertility and mortality, serving as a critical foundation for future demographic predictions.

#### DHS spatial data repository

The 707-district shapefile corresponding to the NFHS-2021 survey was taken from the DHS Spatial Data Repository for India^18^. This shapefile ensures that all clusters that were randomly displaced stay within the district that they were originally assigned to and represent the district boundaries as of March 31^st^, 2017.

#### Andhra Pradesh assembly constituencies (AC) to district linkages

Data was taken from the Chief Electoral Officer (CEO) of Andhra Pradesh which showed the AC to district linkages used to update the 707 DHS district shapefile^19^.

#### Survey of india external country boundary

The official international boundary of India was taken from the Survey of India^20^.

#### Bharatmaps shape files and election commission of india

We obtained access to shapefiles from *Bharatmaps* through an API token specifically created for India Policy Insights^21^. Using this token, we downloaded the PC and AC shapefiles from *Bharatmaps* as the initial source, before making any adjustments. The Election Commission of India delineates and defines PCs across the country based on the 2008 Delimitation Commission.

#### NITI Aayog aspirational district identification

The list of 112 Aspirational Districts launched by NITI Aayog in January 2018 was taken and provided in Supplementary Table 2^22^. These districts were identified as the “most under-developed districts” across India and therefore are identified as areas of concern^23^.

#### Election commission of India PC reservation status

The reservation status of each PC (General, Scheduled Caste, or Scheduled Tribe) was taken from the 2019 General Lok Sabha Election file from Election Commission of India. The list of the 543 PCs and their status are provided in Supplementary Table 3^24^.

### Selection of indicators

The selection of indicators for the IPI initiative was carefully guided by their alignment with global and national policy frameworks. We prioritized indicators commonly used in the population health and social determinants policy spectrum for program design, targeting, monitoring, and evaluation purposes. We relied on a combination of academic, and programmatic documents for the selection of indicators to ensure their policy relevance. More specifically, our selection of indicators was based on District-level NFHS factsheets^25^, India-specific SDGs, and Government of India programs on Health, Nutrition, and Population^26^. For instance, the outcome “Anaemia [Any - All Women]” was selected based on its direct relevance with the objectives of the Government of India’s “Anaemia Mukt Bharat” initiative^27^, and the indicator “Exclusive Breastfeeding for Children under 6 Months” was chosen due to its indirect link to the objectives of the Integrated Child Development Services (ICDS)^28^. This gave us a finite set of 145 indicators spanning several datasets.

However, after aligning these indicators from the data-availability standpoint, we restricted our selection to only nationally representative survey data that provided district-representative samples and overtime comparable outcomes. Hence, we resorted to the fourth and fifth rounds of NFHS conducted in 2015–2016 and 2019–2021, respectively. Therefore, the second level screening and selection of indicators was based on the NFHS 2016 and 2021 India Factsheet and report (IIPS 2016 and 2020), reducing the number of indicators to 131. Following the data-specific refinement process (non-comparable outcomes; data unavailability, no normative direction (sex-ratio), same-indicator variants), 122 indicators were selected for NFHS-2021, and 117 indicators were selected for NFHS-2016. The number of indicators differed slightly across the two time periods due to data-specific limitations on direct comparability.

It is worth noting that some of the indicators were excluded based on their segmentation and variants. For example, indicators like the distribution of public and private hospitals in institutional child births were omitted due to the challenges in presenting both distributional estimates and prevalence data simultaneously. Additionally, indicators lacking a clear normative direction—those without discernible trends of improvement or deterioration over time—were excluded from the final list. An example of this would be family size, which cannot be easily classified as either improving or worsening in a policy-relevant context.

All 122 indicators selected for analysis were dichotomous and coded as either Yes (1) or No (0), ensuring clarity in data representation and result interpretation.

#### Updating geographies and shapefiles (Districts and PCs)

The NFHS-2021 and NFHS-2016 surveys provide direct estimates for 707 districts and 640 districts, respectively, covering 28 States and 8 UTs. We updated the 707-district shapefile provided by the DHS Spatial Data Repository by dissolving ACs in Andhra Pradesh to 26 districts as per the CEO of Andhra Pradesh’s AC to District linkage details. Then, the external boundary of the district shapefile was updated to match the Survey of India’s external boundary for India. No changes were made to the original PC shapefile given by Bharatmaps aside from one minor alignment to align Araku and Eluru PCs.

NFHS-2016 and NFHS-2021 clusters were reassigned to the updated geographic boundaries for nesting within 720 districts and 543 PCs. For districts, the cluster to district linkages that existed in the DHS microdata were taken as is without any adjustments made for all NFHS-2021 clusters except to the clusters that fell in the state of Andhra Pradesh and to all NFHS-2016 clusters where the district remained unchanged from NFHS-2016 to NFHS-2021. This resulted in 694 districts in NFHS-2021 and 564 districts in NFHS-2016 where no adjustments were made to the cluster-to-district linkages. For the clusters that fell into the remaining 26 districts in NFHS-2021 (all in Andhra Pradesh) and the 156 districts (76 parent districts) in NFHS-2016, a spatial join using ArcGIS Pro was used to assign clusters to the updated 720 district geometry. In this process, 54 clusters in NFHS-2016 were not assigned to any district because they did not fall into any eligible changed district that originated from their parent district. Therefore, the final number of clusters included in the district estimates was 30,170 (NFHS-2021) and 28,470 (NFHS-2016).

For PCs, a spatial join using ArcGIS Pro was performed to link clusters in NFHS-2016 and NFHS-2021 to the PC geometry^29^. In this process, a total of 180 (NFHS-2016) and 157 (NFHS-2021) unmatched clusters were not assigned to any PC in the shapefile. Additionally, the spatial join process resulted in 43 (NFHS-2016) and 71 (NFHS-2021) mismatched clusters falling into PCs in a different state than which the microdata identified that cluster falling within. For the 9 states/UTs that only had one PC, where the entire state was itself the PC, we assigned the unmatched and mismatched clusters in these states/UTs to the states/UTs these clusters were assigned to in the microdata. This resulted in a total of 152 (NFHS-2016) and 172 (NFHS-2021) clusters that could not be linked to a PC, therefore resulting in a total of 28,372 (NFHS-2016) and 29,998 (NFHS-2021) clusters included in the PC estimates.

### Estimation

We estimated the prevalence (in %) for 122 indicators across 720 districts and 543 PCs for both NFHS rounds (117 indicators for 2016). Additionally, we computed the change in prevalence between NFHS-2016 and NFHS-2021, along with their rankings and decile positions for each geographic unit.

#### National and state-level estimates

The prevalence estimates were computed for larger geographic units, such as states and the national level. These estimates were derived as population-weighted averages directly from individual datasets, providing a broader perspective on population health and social determinants trends across the country.

#### Precision-weighted cluster-level predicted probabilities

First, using data from NFHS, we computed cluster-level predicted probabilities using a four-level multilevel regression model for both 2016 and 2021. This model accounts for the nested structure of the data, whereby individuals (level-1) are nested within villages or groups of villages referred to as clusters (level-2) in districts (or PCs) (level-3) in states (level-4), allows for partial pooling of information across units, and in doing so produces more stable predictions, especially in geographic areas with small sample sizes.

The model can be written as: ${\rm{logit}}({\pi }_{{ijkl}})={\beta }_{0}+{f}_{l}+{v}_{{kl}}+{u}_{{jkl}}$ where ${\rm{logit}}({\pi }_{{ijkl}})$ denotes the logit of the probability ${\pi }_{{ijkl}}$ or log odds that the indicator is positive for individual $i$ in cluster $j$ in district $k$ in state $l$, and where ${f}_{l}$, ${v}_{{kl}}$, and ${u}_{{jkl}}$ are state, district, and cluster random effects. The random effects are assumed to have normal distributions with zero means and constant variances: ${f}_{l} \sim N(0,{\sigma }_{f}^{2})$, ${v}_{{kl}} \sim N\left(0,{\sigma }_{v}^{2}\right)$, and ${u}_{{jkl}} \sim N\left(0,{\sigma }_{u}^{2}\right)$ where ${\sigma }_{f}^{2}$, ${\sigma }_{v}^{2}$, and ${\sigma }_{u}^{2}$ capture state-level, district-level, and cluster-level variation, respectively, in the prevalence of the indicator.

For model estimation, we first used first-order Marginal Quasi-Likelihood (MQL1) to obtain realistic starting values for the model parameters, followed by Markov Chain Monte Carlo (MCMC) methods for final inference. MCMC estimation enables fully Bayesian inference, providing posterior distributions for all model parameters and allowing robust uncertainty quantification via 95% credible intervals for all model parameters and predicted prevalences^30–32^. All MCMC parameter chains were run with a burn-in period and chain length chosen to ensure convergence and sufficient mixing, typically with 5,000 to 25,000 iterations. Convergence and mixing were judged by inspecting MCMC chain diagnostics and plots and by monitoring the effective sample size (ESS). To improve the MCMC mixing for the cluster-level random effect variance, we used the parameter expansion method. While nearly all parameters in all models achieved ESSs above 250, a few parameters—particularly for rare outcomes or those with limited variation—had lower ESS. These indicators are flagged in the Supplementary Table 4, which summarizes the ESS for all model parameters. The full set of estimation results with standard errors for all 122 indicators is presented in Supplementary Table 5. Multilevel modelling was performed using the STATA 19^33^ and MLwiN 3.13^34^ software program (using *runmlwin*)^35^.

Based on the MCMC estimation, we then generated precision-weighted cluster-level predicted probabilities for all 122 indicators. For more robust estimates, these probabilities were predicted by pooling information (borrowing strength) from other clusters that share the same district membership. Cluster-level predicted probabilities were summarized by the mean values of their posterior distributions, and the 2.5th and 97.5th percentiles were used to compute 95% credible intervals. These cluster-level predictions were then averaged to produce prevalence measures at the district and PC levels. To quantify uncertainty at these higher geographic levels, we computed 95% credible intervals by summarizing the posterior distribution of each district- and PC-level prevalence using the 2.5th and 97.5th percentiles of the aggregated chain values.

It is important to note that the number of clusters across indicators varied, based on the target respondents and hence the sample size (Supplementary Table 6). Further, indicators for those districts and PCs with no cluster sample had to be dropped. This process generated estimates for 29,770 clusters in the KR file for NFHS-2021 and 28,332 clusters for NFHS-2016, 30,161 clusters in the IR file for NFHS-2021 and 28,521 clusters in NFHS-2016, 9,102 clusters in the MR file for NFHS-2021 and 9,887 clusters in NFHS-2016, and 30,170 clusters in the PR file for NFHS-2021 and 28,524 clusters in NFHS-2016.

### Final Estimates/Dataset

#### Prevalence estimates

The district and PC-level prevalence (in %) of each indicator is computed by taking the simple average of the cluster-level probabilities. For example, the prevalence of underweight children in *Karauli* District, *Rajasthan*, was estimated to be 34.1% (95% CI: 29.6; 38.9) for 2021, determined by averaging the predicted probabilities from 44 clusters within the district. This methodology was applied consistently across both NFHS-2016 and NFHS-2021 data to estimate the prevalence for all indicators for all 720 districts and 543 PCs.

#### Headcount estimates

Using the above prevalence estimates for districts and PC, along with the Census population projections, we computed indicator-specific headcounts for all districts and PCs (Supplementary Table 7). For this, we first extracted the weighted share (%) of each district and PC in the NFHS-2021 total sample (indicator-specific). We then computed the total projected population (denominator) for each district and PC using the product of the above-computed sample distribution (from NFHS-2021) and India’s total projected population for 2021 (from the Census of India). Finally, the product of district-level prevalence and denominators gave us the indicator-specific headcount for all districts and PCs. For example, for underweight children aged 0-59 months, if *Kupwara* district accounted for 4.2% of the total NFHS sample for children aged 0-59 months, this percentage was applied to the national population of 114,273,000 children aged 0-59 months, resulting in a projected population of 4,799,466 children (0-59 months) for *Kupwara* in 2021. Headcount estimates were only computed for NFHS-2021 due to data-specific limitations.

#### Outcome-based ranking and positioning of geographies

Further, each geographic unit was ranked based on the prevalence and headcount estimates, where a lower rank indicates a better outcome. The ranks were adjusted for the normative direction of the indicators. For instance, Amritsar District ranked 19th in terms of underweight children with a prevalence of 12.3% (95% CI – 9.1; 15.9), while Aravalli District ranked 700^th^, exhibiting a prevalence rate of 45.3% (95% CI: 39.7; 50.8) for 2021. We also computed the decile positions of each geographic unit for all indicators, with lower deciles indicating better positions.

The change in prevalence from NFHS-2016 to NFHS-2021 is measured by calculating the percentage point difference by subtracting the 2016 prevalence from the 2021 prevalence. Geographic units are categorized into four groups based on the direction and magnitude of change – two indicating improvement, two indicating worsening, with further subdivisions by median cut-offs.

A full summary of national means and the range of prevalence across districts and PCs for all 122 indicators in 2016 and 2021 is presented in Table 1.

**Table 1:** Prevalence estimates for selected indicators, 2021 and 2016.

Indicator No.	Category-Indicator Label	2021 Prevalence (%)			2016 Prevalence (%)		
		National Mean	District Min - Max	PC Min - Max	National Mean	District Min - Max	PC Min - Max
	**Socio-Economic Profile**						
1	Population with BPL cards	45.6	5.2–97.7	3.9–96.9	39.3	2.6–98.2	2.6–93.6
	**Health Care**						
2	Acute Respiratory Infection [All Children]	2.8	0.4–9.1	0.4–8.9	2.7	0.4–18.2	0.4–15.0
3	Acute Respiratory Infection [Children Getting Treatment - Facility]	69.2	22.5–94.2	30.3–94.2	73.2	19.8–95.8	27.6–95.1
4	Diarrhoea [Received ORS]	60.7	37.3–88.0	37.3–84.3	50.7	21.0–92.8	21.8–83.7
5	Diarrhoea [Received Zinc]	31.5	6.8–71.0	9.2–58.4	20.9	2.7–78.9	2.7–66.5
6	Diarrhoea Treatment [Facility]	71.0	26.1–87.7	31.6–86.8	68.0	14.2–92.1	19.5–92.1
7	DPT Vaccination [3 Doses]	86.7	62.4–97.8	70.3–97.5	78.8	17.7–98.2	18.5–98.1
8	Full Vaccination	76.9	45.8–96.3	48.1–96.3	62.6	11.3–96.4	13.9–95.1
9	Full Vaccination [Vaccination Card]	82.6	60.2–97.7	65.6–97.0	77.9	40.1–96.5	48.7–96.4
10	Health Insurance [Any]	40.5	1.4–97.9	2.2–96.8	27.4	0.8–92.1	1.6–87.1
11	Hepatitis B Vaccine [3 Doses]	81.9	36.1–98.0	56.0–97.6	63.5	9.0–97.2	10.8–96.4
12	ICDS Benefits [Children]	67.6	11.3–97.0	22.5–96.2	54.1	6.0–91.9	8.8–92.4
13	Low Birth Weight	17.7	3.6–30.9	4.5–28.3	17.7	3.0–37.2	4.1–34.1
14	Measles-Containing Vaccine [First Dose]	88.2	61.5–98.6	70.1–98.4	81.6	21.6–97.8	31.5–97.4
15	Measles-Containing Vaccine [Second Dose]	59.0	11.9–90.8	15.2–90.3			
16	Polio Vaccination [3 Doses]	80.4	53.6–97.0	56.9–96.6	73.0	27.0–97.5	32.9–97.3
17	Rotavirus Vaccine [3 Doses]	36.9	0.3–97.9	0.3–97.1			
18	Vitamin A Dose	67.8	20.9–94.5	29.7–94.6	62.8	13.5–93.5	20.6–93.1
19	Zero Dose [Child Immunization]	6.7	1.0–23.7	1.2–13.1	10.4	0.7–69.5	0.8–55.7
	**Maternal Health and Family Planning**						
20	Antenatal Care Visit [Four or More]	59.5	4.5–98.4	10.9–97.8	52.1	2.1–98.8	5.0–98.8
21	Antenatal Care Visit [First Trimester]	70.2	26.6–98.6	31.8–98.6	59.0	5.6–97.3	14.3–97.1
22	Birth Registration	89.1	52.9–98.9	55.2–98.9	79.7	25.5–99.4	29.4–99.4
23	Birth Weight Recorded	91.4	36.0–99.7	54.0–99.7	79.1	9.7–99.7	21.2–99.7
24	Caesarean Section Delivery	21.7	1.3–85.1	3.0–79.7	17.4	1.0–81.6	1.6–80.2
25	Caesarean Section in Private Sector	47.8	11.1–92.0	17.0–92.0	41.4	10.1–86.8	11.7–86.0
26	Caesarean Section in Public Sector	14.4	0.9–70.3	1.1–61.6	12.0	0.8–68.4	0.9–54.5
27	Childbirths in Public Facility	62.0	20.2–96.2	21.0–95.5	52.3	8.4–96.0	16.8–93.9
28	Condom	9.4	0.2–42.1	0.2–36.3	5.6	0.1–27.5	0.1–27.1
29	Family Planning [Any Methods by Women]	66.7	12.3–88.8	24.0–87.3	53.5	3.4–85.4	4.2–83.2
30	Family Planning [Modern]	56.4	11.1–81.3	16.6–81.5	47.8	3.4–79.5	4.3–76.9
31	Family Planning [Unmet Need]	9.4	2.0–31.7	2.3–27.3	12.9	2.7–33.1	3.3–30.8
32	Family Planning Services Quality [Family Planning Counselling]	29.7	4.6–70.9	9.4–69.7	19.8	4.2–58.0	5.5–52.7
33	Female Sterilization	37.9	1.8–78.7	3.0–78.7	36.0	1.1–76.1	2.1–76.1
34	Family Planning Services Quality [Side Effects Counselling]	62.3	14.5–96.7	14.5–96.7	46.6	8.8–95.8	8.8–94.9
35	Home Delivery by Skilled Health Personnel	3.2	0.2–18.1	0.2–12.7	4.3	0.5–29.6	0.5–29.1
36	Injectables	0.6	0.0–7.8	0.0–4.6	0.2	0.0–1.6	0.0–1.4
37	Institutional Childbirth	88.6	23.3–99.8	43.9–99.8	78.9	9.7–99.9	35.1–99.9
38	Iron Folic Acid [100 days or More]	44.3	2.2–94.8	6.6–94.8	30.6	1.0–89.9	2.8–89.2
39	Iron Folic Acid [180 days or More]	26.2	1.0–84.4	2.1–83.3	14.6	0.4–78.0	0.4–78.0
40	IUD/PPIUD	2.1	0.1–30.4	0.1–20.2	1.5	0.1–26.6	0.1–22.7
41	Male Sterilization	0.3	0.0–20.2	0.0–11.3	0.3	0.0–12.8	0.0–8.0
42	Maternal Care Quality [Postpartum]	89.8	78.9–99.8	86.6–99.8	89.9	51.7–99.5	60.2–99.5
43	Mother and Child Protection Card	95.9	55.5–99.8	71.3–99.8	89.3	21.3–99.5	28.9–99.4
44	Neonatal Tetanus	92.6	66.1–98.8	81.1–98.8	89.8	30.8–99.1	48.5–99.0
45	Pill	5.1	0.1–52.5	0.1–45.2	4.1	0.1–33.7	0.1–33.1
46	Postnatal Care [Mothers]	80.3	25.6–98.3	38.2–98.1	62.8	5.0–95.3	18.6–95.3
47	Pregnancy Registration	94.0	49.5–99.6	62.0–99.4	85.6	20.5–99.3	22.7–99.1
48	Skilled Birth Attendance	89.6	38.9–99.8	55.1–99.8	81.8	17.9–99.9	40.3–99.9
49	Unmet Need for Spacing	4.1	1.0–22.7	1.1–19.6	5.7	1.3–20.8	1.5–17.1
	**Morbidity and Mortality**						
50	Diarrhoea [Children]	7.3	1.1–36.4	1.7–29.8	9.2	1.7–43.0	2.3–30.0
51	Elevated Blood Pressure or On Medication [Men]	16.3	5.1–39.6	6.2–35.2	14.8	7.4–34.6	7.3–30.8
52	Elevated Blood Pressure or On Medication [Women]	11.7	4.2–29.9	4.5–26.7	11.3	4.3–32.8	4.6–21.8
53	High Blood Sugar [Men]	5.1	1.3–12.0	1.8–10.6	4.2	2.2–6.3	2.3–6.3
54	High Blood Sugar [Women]	4.2	1.4–8.2	1.6–8.2	3.0	1.9–10.8	2.3–10.8
55	High or Very High Blood Sugar or On Medication [Men]	9.8	2.5–19.8	3.1–19.8	9.6	4.7–17.4	5.0–17.4
56	High or Very High Blood Sugar or On Medication [Women]	8.2	2.6–15.6	2.8–15.6	6.5	2.1–13.4	2.7–12.6
57	Mildly Elevated Blood Pressure [Men]	12.0	4.0–30.5	4.7–25.4	11.6	5.0–23.8	5.2–21.2
58	Mildly Elevated Blood Pressure [Women]	7.7	2.8–18.2	3.0–16.3	6.8	2.8–17.4	2.8–14.9
59	Moderate or Severe Blood Pressure [Men]	3.3	0.7–13.2	0.8–9.2	3.6	1.4–9.7	1.4–7.1
60	Moderate or Severe Blood Pressure [Women]	2.2	0.6–9.6	0.6–7.6	2.5	1.0–12.5	1.1–6.8
61	Probability of Dying before Five Years	3.7	0.7–8.2	0.7–7.7	4.4	0.9–9.1	0.9–9.0
62	Probability of Dying before One Year	3.4	0.6–6.9	0.6–6.6	3.9	0.8–7.3	0.8–7.3
63	Probability of Dying within 28 Days	2.5	0.5–5.0	0.5–4.8	2.9	0.8–7.6	0.8–8.4
64	Risky Waist-to-hip Ratio [Women]	58.4	20.1–96.6	23.5–91.3			
65	Very High Blood Sugar [Men]	4.0	0.6–9.5	0.8–9.2	4.5	1.8–8.7	2.0–8.4
66	Very High Blood Sugar [Women]	3.3	0.6–7.9	0.8–7.9	2.8	0.8–6.4	0.9–6.0
	**Nutrition [Clinical/Anthropometry**]						
67	Anaemia [Any - Adolescent Women]	59.1	20.6–92.7	21.8–91.8	54.1	11.1–85.0	19.2–81.2
68	Anaemia [Any - All Women]	53.8	16.1–91.3	24.3–90.7	53.1	12.7–84.2	21.9–79.9
69	Anaemia [Any - Pregnant Women]	49.7	14.5–74.4	20.3–73.9	50.4	15.7–74.2	17.5–71.1
70	Child Anaemia [Any]	68.0	22.4–93.6	30.4–92.4	58.7	7.7–92.7	18.7–89.5
71	Child Stunting	35.5	15.1–56.5	17.0–51.7	38.4	15.1–62.7	15.2–62.7
72	Child Underweight	32.1	9.3–59.2	11.8–54.8	35.8	8.4–64.6	11.4–61.0
73	Child Wasting	19.2	6.8–43.9	7.1–32.6	21.0	3.9–43.6	6.0–39.5
74	Mild Anaemia [Children]	29.2	17.7–43.5	19.6–39.6	27.9	8.1–39.4	15.0–37.6
75	Mild Anaemia [Women]	24.2	12.0–33.5	13.5–33.5	24.7	11.7–57.4	19.6–57.4
76	Moderate Anaemia [Children]	36.5	6.3–62.3	8.7–60.2	29.3	2.1–64.7	4.9–60.5
77	Moderate Anaemia [Women]	27.8	5.6–69.1	9.2–67.8	26.6	3.7–61.5	6.1–53.9
78	Overweight Children	3.4	0.7–15.6	0.9–9.8	2.1	0.3–9.1	0.3–6.7
79	Overweight or Obese [Women]	23.9	4.1–52.6	4.5–52.6	20.6	2.5–47.5	3.8–47.5
80	Severe Anaemia [Children]	2.1	0.3–6.9	0.3–6.5	1.5	0.2–7.0	0.3–6.0
81	Severe Anaemia [Women]	2.5	0.4–32.5	0.6–25.8	2.4	0.3–20.2	0.3–11.6
82	Severe Stunting [Children]	15.3	4.6–28.7	4.8–26.2	16.3	4.1–39.4	4.3–35.9
83	Severe Underweight [Children]	10.3	2.3–26.6	3.1–25.1	10.8	1.6–31.1	2.2–26.4
84	Severe Wasting [Children]	7.6	2.1–28.0	2.1–18.6	7.5	1.3–25.2	1.7–18.7
85	Underweight [Women]	18.7	2.2–41.5	3.2–36.6	22.9	3.7–45.9	5.7–45.8
	**Nutrition [Diet]**						
86	Adequate Diet [Breastfed Children]	10.6	2.7–31.7	2.7–29.1	8.7	1.3–29.9	1.3–28.0
87	Adequate Diet [Non-breastfed Children]	12.5	3.3–32.1	3.3–26.2	14.5	1.7–60.8	1.8–60.7
88	Adequate Diet [Total]	10.9	2.8–35.1	2.8–28.2	9.6	1.5–35.9	1.5–34.7
89	Early Breastfeeding Initiation	42.2	7.3–91.2	9.1–87.4	42.3	12.2–88.8	12.5–82.0
90	ICDS Supplementary Nutrition	69.3	9.9–99.2	16.8–98.7	56.0	4.6–97.1	7.2–95.5
91	Iodized Salt Intake	93.9	39.5–98.7	43.3–99.1	92.9	44.6–100.0	44.8–99.9
92	Exclusive Breastfeeding [Under 6 Months]	63.4	42.2–86.1	48.1–84.7	54.9	17.2–84.7	18.3–83.6
93	Receiving Solid/Semi–solid Food [6–8 Months]	46.0	20.7–83.5	21.1–79.7	42.7	14.9–83.0	15.5–80.7
94	Zero Food [Children]	17.8	3.7–45.2	3.7–45.0	17.2	4.0–33.5	4.7–30.5
	**Social Infrastructure**						
95	Access to Electricity	96.8	66.1–100.0	61.0–100.0	88.0	30.8–100.0	31.7–99.9
96	Clean Cooking Fuel	56.0	6.5–99.9	6.2–99.8	41.2	1.1–99.9	1.9–99.7
97	Death Registration	70.7	15.7–99.2	23.1–99.2			
98	Handwashing Facilities	72.8	13.8–99.4	15.5–99.5	61.1	12.8–99.6	15.1–98.0
99	Hygienic Protection Methods [Menstruation]	77.6	30.0–99.4	35.2–99.3	57.9	12.7–98.6	15.2–97.3
100	Improved Sanitation Facility	70.0	27.3–99.8	10.7–99.8	48.4	4.8–99.8	8.1–99.8
101	Improved Source of Drinking Water	95.9	41.6–100.0	43.7–100.0	90.2	24.4–100.0	37.4–100.0
102	Internet Usage [Women]	33.3	7.2–83.5	8.5–78.5			
103	Private Latrine	72.5	32.1–99.8	11.4–99.9	51.1	3.2–99.7	11.3–99.8
104	Safe Stool Disposal	39.3	3.6–94.0	4.3–94.1	36.1	1.4–99.5	3.8–98.8
105	Women with Personal Mobile Phone	54.9	22.9–92.3	25.4–92.3	45.8	12.8–88.8	17.7–87.1
106	Alcohol Consumption [Men]	17.5	0.5–63.0	0.6–53.5	29.2	1.5–83.1	1.7–69.1
107	Alcohol Consumption [Women]	0.7	0.5–39.4	0.5–31.5	1.2	0.0–50.9	0.0–31.2
108	Child Marriage [Boy]	18.2	3.0–50.7	3.1–42.1	20.1	3.3–49.5	3.3–44.8
109	Child Marriage [Girl]	23.6	2.3–57.9	2.9–58.8	26.7	2.8–59.1	2.9–58.5
110	Currently Working Women	25.0	4.9–65.6	4.9–57.9	24.4	5.4–65.3	5.1–60.5
111	Female School Attendance	71.8	45.1–98.4	49.4–98.0	69.2	36.8–98.4	40.7–98.3
112	High School Matriculation [Men]	50.2	15.7–84.8	22.1–82.5	45.9	17.4–77.6	20.5–77.2
113	High School Matriculation [Women]	41.0	13.3–87.5	14.5–86.3	35.7	9.5–86.0	10.7–82.9
114	Intimate Partner Violence [Against Women]	29.4	3.3–67.7	4.1–65.9	31.5	1.9–62.8	2.2–59.0
115	Literacy [Men]	84.4	47.9–98.2	58.0–98.2	84.4	51.5–98.9	59.9–98.8
116	Literacy [Women]	71.4	36.6–98.9	38.3–98.8	68.4	26.0–99.2	31.1–99.2
117	Population below 15 Years	26.5	16.3–49.7	16.0–42.9	28.6	16.6–43.6	16.7–42.5
118	Sexual Violence [Young Women]	1.7	0.2–3.9	0.2–3.9	2.1	0.3–9.0	0.3–7.9
119	Teenage Pregnancy	6.8	0.6–24.5	0.7–24.1	7.9	1.5–25.9	1.6–26.1
120	Tobacco Consumption [Women]	4.1	0.0–64.5	0.0–54.3	6.8	0.1–78.9	0.1–67.6
121	Tobacco Use [Men]	32.6	6.5–79.7	7.4–74.6	44.5	12.9–84.1	14.2–80.4
122	Women Participation in Household Decisions	88.7	57.4–99.4	77.9–99.3	83.9	59.8–98.4	63.3–97.7

*Note*. The following indicators were excluded from 2016 estimates as the indicator was not available for 2016: Measles-Containing Vaccine (2nd Dose), Rotavirus Vaccine (3 Doses), Risky Waist-to-Hip Ratio (Women), Death Registration, Internet Usage (Women).

## Data Records

### Data set

The India Policy Insights Open Dataset^36^ is available at 10.7910/DVN/7QB1BY. The final ready-to-use dataset is in Excel (.xlsx) format, namely, IPI_District_Data.xlsx and IPI_PC_Data.xlsx for districts and PCs respectively.

Each of the data files contains five spreadsheets. The first spreadsheet *“Sheet & Column Description”* contains information and descriptions about all the sheets and columns in the dataset. We have assigned numeric identifiers (IDs) to the variables, like indicators, categories, states, districts, and PCs. The second spreadsheet (“*Label Dictionary*”) depicts information on labels to these assigned IDs. The third spreadsheet contains All-India estimates (prevalence, headcount, and change), for all the indicators. The fourth sheet (“*Indicator-State Data*”) reflects the state-level final estimates for all indicators. Finally, the fifth spreadsheet (“*Indicator-District Data*” and “*Indicator-PC Data*”) has the district and PC-level final estimates.

## Technical Validation

Several quality checks, validation processes, and sensitivity tests were implemented to ensure these estimates’ accuracy and reliability. Validation checks were conducted at multiple stages before and after the precision-weighted estimates were performed. Weighted prevalence estimates, calculated from individual data, were cross-referenced with figures from the NFHS India Report to confirm the accuracy of indicator calculations. Supplementary Table 8 compares our all-India estimates with the NFHS Report for India. While for most of the indicators, we were able to verify our estimate with the NFHS report or factsheet with no or negligible difference, for a few indicators, verification of estimated prevalence was not possible due to incomparability in the reference sample. For example, the reference age criteria for the indicator “Tobacco Use [Men]” was 15+ years as per the NFHS Report; however, our estimation of the same indicator was done by taking the age reference age group of 15-49 years. Additional descriptive statistical measures, such as the mean, median, interquartile range (IQR), and histograms, were employed to evaluate the distribution of outcomes, and these were compared with untreated figures. Furthermore, District and PC-level denominators (total population of the corresponding indicator) were carefully compared against the 2011 Census data to ensure the precision of headcount estimations. All final estimates were subjected to a thorough review by a quality assurance team to identify and correct any potential errors or miscalculations.

The estimation procedure was based on the following underlying assumptions. The multilevel model assumes random effects to be normally distributed (on the log-odds scale), and the outcome is assumed to follow a binomial (logistic) distribution at the individual level. Further, the model, while allowing for the most important spatial correlations in the data, nevertheless still makes simplifying assumptions. Additionally, while reassigning clusters to updated 720 district boundaries, it was assumed that the aspect of “district representativeness” remains unaffected for NFHS-2016 estimates for the 115 districts that were carved out of a single existing district. Finally, for computing headcount estimates for 2021, the sample distribution across population sub-groups and geographies (districts and PCs) is assumed to reflect the actual population distribution.

The following limitations need to be acknowledged for careful interpretation of the estimates. First, it is worth noting that the COVID-19 pandemic may have introduced disruptions that could influence responses or data collection processes. However, due to the limitations of the survey dataset, such as the absence of specific indicators capturing pandemic-related impacts, we were unable to directly account for or isolate the effects of COVID-19 in our analysis. We recommend that future research explore this dimension more explicitly using data specifically designed to capture pandemic-related effects. Second, the following model-specific limitations warrant mention. For a few outcomes, district- and PC-level predictions may suffer from shrinkage bias, with predictions pulled towards the national mean for districts or PCs with very small sample sizes or high uncertainty. However, given that the NFHS 2016 and 2021 provided a district-representative sample for all the selected outcomes, our model estimates (for both districts and PC) are reliable in terms of the aforementioned biases.

## Usage Notes

Our dataset and accompanying visualization tool^37^ will be immediately useful for administrators and their research teams, as well as journalists, civic organizations, students, and academics. This dataset, the first of its kind, spans two vital sub-national units: districts, essential for development administration, and parliamentary constituencies, representing populations through elected officials. It is robust for assessments at the population level. However, its use for launching specific programs in districts or PCs should be preceded by a targeted survey.

## Supplementary information

Supplementary File

## Data Availability

The India Policy Insights dataset is publicly available at 10.7910/DVN/7QB1BY.

## Code availability

A sample code for Indicator 1 (NFHS-2021) is provided in Appendix A of the supplementary file. The complete set of scripts used for data preparation, model estimation, and post-processing across all 122 indicators is available from the corresponding authors upon request.
