# Supplementary material for: India Policy Insights: Estimates of Population Health and Social Determinants Indicators Across Policy Units
Source: Sci Data. 2025 Sep 30;12:1592. doi: 10.1038/s41597-025-05923-8 (PMC12484729; doi:10.1038/s41597-025-05923-8)
Supplement: Supplementary file 1 — Supplementary File [file 41597_2025_5923_MOESM1_ESM.docx]

**Supplementary File** **for “India Policy Insights: Estimates of Population Health and Social Determinants Indicators Across Policy Units”**

**Table of Contents**

**Supplementary Table 1. Definitions for 122 Indicators**

**Supplementary Table 2. List of 112 Aspirational Districts**

**Supplementary Table 3. List of 543 Parliamentary Constituencies and their Reservation Status**

**Supplementary Table 4. Effective Sample Sizes for 122 Indicators, 2021 and 2016**

**Supplementary Table 5. Multilevel Model Estimates for 122 Indicators, 2021 and 2016**

**Supplementary Table 6. Sample Sizes for 122 Indicators, 2021 and 2016**

**Supplementary Table 7. Headcount Estimates for 122 Indicators, 2021 and 2016**

**Supplementary Table 8. Estimated and Reported Prevalence for Validation**

**Appendix A. Sample Code for Indicator 1: Population with Below Poverty Line Cards, 2021**

**Supplementary Table 1.** **Indicator Definitions and Category Labels**

| **Indicator No** | **Category-Indicator Label** | **Definition of the Indicator and Study Population** |
| --- | --- | --- |
|  | **Socio-Economic Profile** |  |
| 1 | Population with BPL cards | Percentage of the sample population self-reported as having a Below Poverty Line (BPL) card |
|  | **Health Care** |  |
| 2 | Acute Respiratory Infection [All Children] | Percentage of children (under age 5 years) with symptoms of Acute Respiratory Infection (ARI) in the 2 weeks preceding the survey |
| 3 | Acute Respiratory Infection [Children Getting Treatment - Facility] | Percentage of children (under age 5 years) with fever or symptoms of Acute Respiratory Infection (ARI) in the 2 weeks preceding the survey who were taken to a health facility or health provider |
| 4 | Diarrhoea [Received ORS] | Percentage of children who received an oral rehydration solution (ORS) packet among those who had diarrhoea in the 2 weeks preceding the survey and sought medical care |
| 5 | Diarrhoea [Received Zinc] | Percentage of children who were given zinc among those who had diarrhoea in the 2 weeks preceding the survey and sought medical care |
| 6 | Diarrhoea Treatment [Facility] | Percentage of children (under age 5 years) who had diarrhoea in the 2 weeks preceding the survey and were taken to a health facility or health provider |
| 7 | DPT Vaccination [3 Doses] | Percentage of children (12-23 months) who received 3 doses of diphtheria, pertussis (whooping cough), and tetanus (DPT) vaccine |
| 8 | Full Vaccination | Percentage of children (12-23 months) who received full vaccination based on information from either a vaccination card or mother's recall |
| 9 | Full Vaccination [Vaccination Card] | Percentage of children (12-23 months) fully vaccinated based on information on vaccination card |
| 10 | Health Insurance [Any] | Percentage of the sample population covered under any health insurance scheme (fully or partly) |
| 11 | Hepatitis B Vaccine [3 Doses] | Percentage of children (12-23 months) who received 3 doses of Hepatitis B vaccine |
| 12 | ICDS Benefits [Children] | Percentage of children who received benefits from the Integrated Child Development Scheme (ICDS) in the last twelve months |
| 13 | Low Birth Weight | Percentage of alive births reporting a birth weight of less than 2.5 kg |
| 14 | Measles-Containing Vaccine [First Dose] | Percentage of children (12-23 months) who received their first dose of measles-containing vaccine (MCV) |
| 15 | Measles-Containing Vaccine [Second Dose] | Percentage of children (24-35 months) who received a second dose of measles-containing vaccine (MCV) |
| 16 | Polio Vaccination [3 Doses] | Percentage of children (12-23 months) who received 3 doses of polio vaccine |
| 17 | Rotavirus Vaccine [3 Doses] | Percentage of children (12-23 months) who received 3 doses of rotavirus vaccine |
| 18 | Vitamin A Dose | Percentage of children (9-35 months) who received a Vitamin A dose in the last six months |
| 19 | Zero Dose [Child Immunization] | Percentage of children (12-23 months) who did not receive the first dose of diphtheria, pertussis (whooping cough), and tetanus (DPT) vaccine |
|  | **Maternal Health and Family Planning** |  |
| 20 | Antenatal Care Visit [Four or More] | Percentage of women (15-49 years) who have received four or more antenatal care (ANC) check-ups for their most recent birth |
| 21 | Antenatal Care Visit [First Trimester] | Percentage of women (15-49 years) who had their first antenatal care (ANC) visit during the first trimester of their pregnancy for their most recent birth |
| 22 | Birth Registration | Percentage of live births in the 5 years preceding the survey registered with the civil authority |
| 23 | Birth Weight Recorded | Percentage of live births in the 5 years preceding the survey that had either a written record of the child's weight at the time of birth or the mother was able to recall the weight at the time of birth |
| 24 | Caesarean Section Delivery | Percentage of institutional childbirths delivered by Caesarean section |
| 25 | Caesarean Section in Private Sector | Percentage of births in a private health facility that were delivered by caesarean section |
| 26 | Caesarean Section in Public Sector | Percentage of births in a public health facility that were delivered by caesarean section |
| 27 | Childbirths in Public Facility | Percentage of institutional childbirths in public health facilities |
| 28 | Condom | Percentage of currently married women who use condoms as family planning method |
| 29 | Family Planning [Any Methods by Women] | Percentage of currently married women who use any of the family planning methods |
| 30 | Family Planning [Modern] | Percentage of currently married women (15-49 years) using modern methods of family planning |
| 31 | Family Planning [Unmet Need] | Percentage of currently married women (15-49 years) who have unmet needs for family planning |
| 32 | Family Planning Services Quality [Family Planning Counselling] | Percentage of female non-users of family planning who were counselled about family planning by a health worker |
| 33 | Female Sterilization | Percentage of female users who were ever counselled about side effects of current family planning methods |
| 34 | Family Planning Services Quality [Side Effects Counselling] | Percentage of currently married women (15-49 years) who have undergone sterilization |
| 35 | Home Delivery by Skilled Health Personnel | Percentage of home births by skilled health personnel |
| 36 | Injectables | Percentage of currently married women who use injectables as a family planning method |
| 37 | Institutional Childbirth | Percentage of childbirths delivered in an institutional facility |
| 38 | Iron Folic Acid [100 days or More] | Percentage of mothers who consumed iron folic acid for 100 days or more during their most recent pregnancy |
| 39 | Iron Folic Acid [180 days or More] | Percentage of mothers who consumed iron folic acid for 180 days or more during their most recent pregnancy |
| 40 | IUD/PPIUD | Percentage of currently married women who use an Intrauterine Device (IUD) or Postpartum Intrauterine Device (PPIUD) as a family planning method |
| 41 | Male Sterilization | Percentage of men (15-49 years) who have undergone sterilization |
| 42 | Maternal Care Quality [Postpartum] | Percentage of women ever checked by a health professional during their stay in the facility after their delivery |
| 43 | Mother and Child Protection Card | Percentage of registered pregnancies for which the mother received a Mother and Child Protection (MCP) card |
| 44 | Neonatal Tetanus | Percentage of mothers whose last birth was protected against neonatal tetanus |
| 45 | Pill | Percentage of currently married women who use pills as a family planning method |
| 46 | Postnatal Care [Mothers] | Percentage of mothers who received postnatal care from a doctor/nurse/LHV/ANM/midwife/other health personnel within 2 days of delivery |
| 47 | Pregnancy Registration | Percentage of women (15-49 years) who registered the pregnancy for their most recent birth |
| 48 | Skilled Birth Attendance | Percentage of home deliveries attended by an SBA (Skilled Birth Attendance) trained health worker, out of total home deliveries |
| 49 | Unmet Need for Spacing | Percentage of currently married women (15-49 years) who have unmet needs for birth spacing |
|  | **Morbidity and Mortality** |  |
| 50 | Diarrhoea [Children] | Percentage of children (0-59 months) who had diarrhoea in the 2 weeks preceding survey |
| 51 | Elevated Blood Pressure or On Medication [Men] | Percentage of men (15-49 years) who have elevated blood pressure (systolic > 140 mmHg and/or diastolic > 90 mm of Hg) or are taking medicine to control blood pressure |
| 52 | Elevated Blood Pressure or On Medication [Women] | Percentage of women (15-49 years) who have elevated blood pressure (systolic > 140 mmHg and/or diastolic > 90 mm of Hg) or are taking medicine to control blood pressure |
| 53 | High Blood Sugar [Men] | Percentage of men (15-49 years) whose blood sugar is greater than 140 mg/dl |
| 54 | High Blood Sugar [Women] | Percentage of women (15-49 years) whose blood sugar is greater than 140 mg/dl |
| 55 | High or Very High Blood Sugar or On Medication [Men] | Percentage of men (15-49 years) who have a random blood sugar test greater than 140 mg/dl or are taking medicine to control their blood sugar level at the time of the survey |
| 56 | High or Very High Blood Sugar or On Medication [Women] | Percentage of women (15-49 years) who have a random blood sugar test greater than 140 mg/dl or are taking medicine to control their blood sugar level at the time of the survey |
| 57 | Mildly Elevated Blood Pressure [Men] | Percentage of men (15-49 years) who have mildly high blood pressure (systolic 140-159 mm of Hg and/or diastolic 90-99 mm of Hg) |
| 58 | Mildly Elevated Blood Pressure [Women] | Percentage of women (15-49 years) who have mildly high blood pressure (systolic 140-159 mm of Hg and/or diastolic 90-99 mm of Hg) |
| 59 | Moderate or Severe Blood Pressure [Men] | Percentage of men (15-49 years) who have severely high blood pressure (systolic > 160 mm of Hg and/or diastolic > 100 mm of Hg |
| 60 | Moderate or Severe Blood Pressure [Women] | Percentage of women (15-49 years) who have severely high blood pressure (systolic > 160 mm of Hg and/or diastolic > 100 mm of Hg) |
| 61 | Probability of Dying before Five Years | Probability of deaths of children (under age 5 years) per 1,000 live births. |
| 62 | Probability of Dying before One Year | Probability of infant deaths (under 1 year) per 1,000 live births. |
| 63 | Probability of Dying within 28 Days | Probability of deaths of children within 28 days of birth per 1,000 live births |
| 64 | Risky Waist-to-hip Ratio [Women] | Percentage of women (15-49 years) who have a high-risk waist-to-hip ratio (>=0.85) |
| 65 | Very High Blood Sugar [Men] | Percentage of men (15-49 years) whose blood sugar is greater than 160 mg/dl |
| 66 | Very High Blood Sugar [Women] | Percentage of women (15-49 years) whose blood sugar is greater than 160 mg/dl |
|  | **Nutrition [Clinical/Anthropometry]** |  |
| 67 | Anaemia [Any - Adolescent Women] | Percentage of women (15-19 years) whose blood haemoglobin level is less than 12 g/dl |
| 68 | Anaemia [Any - All Women] | Percentage of all women (15-49 years) whose blood haemoglobin level is less than 12.0 g/dl for non-pregnant women and less than 11.0 g/dl for pregnant women |
| 69 | Anaemia [Any - Pregnant Women] | Percentage of pregnant women whose blood haemoglobin level is less than 11.0 g/dl |
| 70 | Child Anaemia [Any] | Percentage of children (6-59 months) whose blood haemoglobin level is less than 11.0 g/dl |
| 71 | Child Stunting | Percentage of children (under age 5 years) who are stunted. Stunting is defined as children whose height-for-age z-scores is below -2 SD of WHO Child Growth Standards |
| 72 | Child Underweight | Percentage of children (under age 5 years) who are underweight. Underweight is defined as children whose weight-for-age z-scores is below -2 SD of WHO Child Growth Standards |
| 73 | Child Wasting | Percentage of children (under age 5 years) who are wasted. Wasting is defined as children whose weight-for-height z-scores is below -2 SD of WHO Child Growth Standards |
| 74 | Mild Anaemia [Children] | Percentage of children (6-59 months) whose blood haemoglobin level is between 10g/dl and 11 g/dl |
| 75 | Mild Anaemia [Women] | Percentage of women (15-49 years) whose blood haemoglobin level is between 10g/dl and 11 g/dl (11g/dl and 12 g/dl for pregnant women) |
| 76 | Moderate Anaemia [Children] | Percentage of children (6-59 months) whose blood haemoglobin level is between 7 g/dl and 10 g/dl |
| 77 | Moderate Anaemia [Women] | Percentage of women (15-49 years) whose blood haemoglobin level is between 7 g/dl and 10 g/dl |
| 78 | Overweight Children | Percentage of children (under 5 years) who are overweight |
| 79 | Overweight or Obese [Women] | Percentage of women (15-49 years) whose Body Mass Index (BMI) is greater than or equal to 25.0 kg/m2 |
| 80 | Severe Anaemia [Children] | Percentage of children (6-59 months) whose blood haemoglobin level is less than 7 g/dl |
| 81 | Severe Anaemia [Women] | Percentage of women (15-49 years) whose blood haemoglobin level is less than 7 g/dl |
| 82 | Severe Stunting [Children] | Percentage of children (under age 5 years) who are severely stunted. Severe stunting is defined as children whose height-for-age z-scores is below -3 SD of WHO Child Growth Standards |
| 83 | Severe Underweight [Children] | Percentage of children (under age 5 years) who are severely underweight. Severe underweight is defined as children whose weight-for-age z-scores is below -3 SD of WHO Child Growth Standards |
| 84 | Severe Wasting [Children] | Percentage of children (under 5 years) who have Severe Acute Malnutrition. Severe Acute Malnutrition (SAM) is defined as children whose weight-for-height z-scores is below -3 SD of WHO Child Growth Standards |
| 85 | Underweight [Women] | Percentage of women (15-49 years) whose Body Mass Index (BMI) is less than 18.5 kg/m2 |
|  | **Nutrition [Diet]** |  |
| 86 | Adequate Diet [Breastfed Children] | Percentage of breastfeeding children (6-23 months) receiving an adequate diet |
| 87 | Adequate Diet [Non-breastfed Children] | Percentage of non-breastfeeding children (6-23 months) receiving an adequate diet |
| 88 | Adequate Diet [Total] | Percentage of children (6-23 months) receiving an adequate diet |
| 89 | Early Breastfeeding Initiation | Percentage of children (under age 3 years) who were breastfed within one hour of birth |
| 90 | ICDS Supplementary Nutrition | Percentage of pregnant women regularly taking supplementary nutrition under the Integrated Child Development Scheme (ICDS) program |
| 91 | Iodized Salt Intake | Percentage of sample population consuming iodized salt |
| 92 | Exclusive Breastfeeding [Under 6 Months] | Percentage of infants under 6 months who were exclusively breastfed |
| 93 | Receiving Solid/Semi-solid Food [6-8 Months] | Percentage of children aged 6 to 8 months who received solid/semi-solid food preceding the night before survey |
| 94 | Zero Food [Children] | Percentage of children aged 6-23 months who did not receive any food preceding the night before survey |
|  | **Social Infrastructure** |  |
| 95 | Access to Electricity | Percentage of sample population in households with access to electricity |
| 96 | Clean Cooking Fuel | Percentage of the sample population with access to clean fuel for cooking (electricity, LPG/natural gas, biogas) |
| 97 | Death Registration | Percentage of deaths in the last 3 years registered with the Civil Authority |
| 98 | Handwashing Facilities | Percentage of the sample population with access to handwashing facilities |
| 99 | Hygienic Protection Methods [Menstruation] | Percentage of women (15-49 years) using hygienic methods for protection during their menstrual period (locally prepared napkins, sanitary napkins, tampons, and menstrual cups) |
| 100 | Improved Sanitation Facility | Percentage of sample population with access to improved sanitation facilities (flush or pour-flush to piped sewer system, septic tank or pit latrine, ventilated improved pit latrine, pit latrine with slab, composting toilet). Sanitation facilities are not considered improved when shared with other sample populations, or open to public use |
| 101 | Improved Source of Drinking Water | Percentage of sample population with an improved drinking-water source (piped water into dwelling/yard/plot, piped to a neighbour, public tap/standpipe, tube well or borehole, protected dug well, protected spring, rainwater, tanker truck, cart with a small tank, bottled water, community RO plant) |
| 102 | Internet Usage [Women] | Percentage of women (15-49 years) who have ever used the Internet |
| 103 | Private Latrine | Percentage sample population with access to an individual self-owned latrine |
| 104 | Safe Stool Disposal | Percentage of women practising safe stool disposal |
| 105 | Women with Personal Mobile Phone | Percentage of women (15-49 years) who have a mobile phone for personal use |
| 106 | Alcohol Consumption [Men] | Percentage of men (15 years or above) who consume alcohol |
| 107 | Alcohol Consumption [Women] | Percentage of women (15 years or above) who consume alcohol |
| 108 | Child Marriage [Boy] | Percentage of men (21-25 years) who married before the age of 21 |
| 109 | Child Marriage [Girl] | Percentage of women (20-24 years) who married before the age of 18 |
| 110 | Currently Working Women | Percentage of women (15-49 years) who have worked in the last 12 months and were paid in cash |
| 111 | Female School Attendance | Percentage of the female population age 6 years and above who ever attended school |
| 112 | High School Matriculation [Men] | Percentage of men (20-49 years) who have completed their high school education |
| 113 | High School Matriculation [Women] | Percentage of women (20-49 years) who have completed their high school education |
| 114 | Intimate Partner Violence [Against Women] | Percentage of ever-married women (18-49 years) who have ever experienced spousal violence (physical or sexual) |
| 115 | Literacy [Men] | Percentage of men (15-49 years) who are literate |
| 116 | Literacy [Women] | Percentage of women (15-49 years) who are literate |
| 117 | Population below 15 Years | Percentage of the population below 15 years of age |
| 118 | Sexual Violence [Young Women] | Percentage of women (18-29 years) who experienced sexual violence by age 18 |
| 119 | Teenage Pregnancy | Percentage of women (15-19 years) who were already mothers or pregnant at the time of the survey |
| 120 | Tobacco Consumption [Women] | Percentage of women (15-49 years) who consume any kind type of tobacco |
| 121 | Tobacco Use [Men] | Percentage of men (15-49 years) who consume any kind type of tobacco |
| 122 | Women Participation in Household Decisions | Percentage of currently married women (15-49 years) who usually participate in their household decisions |

**Supplementary Table 2. List of 112 Aspirational Districts**

| **State Name** | **District Name** |
| --- | --- |
| Andhra Pradesh | Alluri Sitharamaraju |
| Andhra Pradesh | Parvathipuram Manyam |
| Andhra Pradesh | Y.S.R. Kadapa |
| Arunachal Pradesh | Namsai |
| Assam | Baksa |
| Assam | Barpeta |
| Assam | Darrang |
| Assam | Dhubri |
| Assam | Goalpara |
| Assam | Hailakandi |
| Assam | Udalguri |
| Bihar | Araria |
| Bihar | Aurangabad |
| Bihar | Banka |
| Bihar | Begusarai |
| Bihar | Gaya |
| Bihar | Jamui |
| Bihar | Katihar |
| Bihar | Khagaria |
| Bihar | Muzaffarpur |
| Bihar | Nawada |
| Bihar | Purnea |
| Bihar | Sheikhpura |
| Bihar | Sitamarhi |
| Chhattisgarh | Bastar |
| Chhattisgarh | Bijapur |
| Chhattisgarh | Dantewada |
| Chhattisgarh | Kanker |
| Chhattisgarh | Kondagaon |
| Chhattisgarh | Korba |
| Chhattisgarh | Mahasamund |
| Chhattisgarh | Narayanpur |
| Chhattisgarh | Rajnandgaon |
| Chhattisgarh | Sukma |
| Gujarat | Dahod |
| Gujarat | Narmada |
| Haryana | Mewat |
| Himachal Pradesh | Chamba |
| Jammu & Kashmir | Baramula |
| Jammu & Kashmir | Kupwara |
| Jharkhand | Bokaro |
| Jharkhand | Chatra |
| Jharkhand | Dumka |
| Jharkhand | Garhwa |
| Jharkhand | Giridih |
| Jharkhand | Godda |
| Jharkhand | Gumla |
| Jharkhand | Hazaribag |
| Jharkhand | Khunti |
| Jharkhand | Latehar |
| Jharkhand | Lohardaga |
| Jharkhand | Pakur |
| Jharkhand | Palamu |
| Jharkhand | Pashchimi Singhbhum |
| Jharkhand | Purbi Singhbhum |
| Jharkhand | Ramgarh |
| Jharkhand | Ranchi |
| Jharkhand | Sahibganj |
| Jharkhand | Simdega |
| Karnataka | Raichur |
| Karnataka | Yadgir |
| Kerala | Wayanad |
| Madhya Pradesh | Barwani |
| Madhya Pradesh | Chhatarpur |
| Madhya Pradesh | Damoh |
| Madhya Pradesh | Guna |
| Madhya Pradesh | Khandwa |
| Madhya Pradesh | Rajgarh |
| Madhya Pradesh | Singrauli |
| Madhya Pradesh | Vidisha |
| Maharashtra | Gadchiroli |
| Maharashtra | Nandurbar |
| Maharashtra | Osmanabad |
| Maharashtra | Washim |
| Manipur | Chandel |
| Meghalaya | Ribhoi |
| Mizoram | Mamit |
| Nagaland | Kiphire |
| Odisha | Balangir |
| Odisha | Dhenkanal |
| Odisha | Gajapati |
| Odisha | Kalahandi |
| Odisha | Kandhamal |
| Odisha | Koraput |
| Odisha | Malkangiri |
| Odisha | Nabarangapur |
| Odisha | Nuapada |
| Odisha | Rayagada |
| Punjab | Ferozepur |
| Punjab | Moga |
| Rajasthan | Baran |
| Rajasthan | Dholpur |
| Rajasthan | Jaisalmer |
| Rajasthan | Karauli |
| Rajasthan | Sirohi |
| Sikkim | Soreng |
| Tamil Nadu | Ramanathapuram |
| Tamil Nadu | Virudhunagar |
| Telangana | Asifabad |
| Telangana | Bhadradri-Kothagudem |
| Telangana | Bhupalpally |
| Tripura | Dhalai |
| Uttar Pradesh | Bahraich |
| Uttar Pradesh | Balrampur |
| Uttar Pradesh | Chandauli |
| Uttar Pradesh | Chitrakoot |
| Uttar Pradesh | Fatehpur |
| Uttar Pradesh | Shravasti |
| Uttar Pradesh | Siddharthnagar |
| Uttar Pradesh | Sonbhadra |
| Uttarakhand | Haridwar |
| Uttarakhand | Udham Singh Nagar |

**Supplementary Table 3. List of 543 Parliamentary Constituencies and their Reservation Status**

| **State Name** | **PC Name** | **Reservation Status** |
| --- | --- | --- |
| Telangana | Adilabad | ST |
| Uttar Pradesh | Agra | SC |
| Gujarat | Ahmedabad East | GEN |
| Gujarat | Ahmedabad West | SC |
| Maharashtra | Ahmednagar | GEN |
| Rajasthan | Ajmer | GEN |
| Uttar Pradesh | Akbarpur | GEN |
| Maharashtra | Akola | GEN |
| Kerala | Alappuzha | GEN |
| Kerala | Alathur | SC |
| Uttar Pradesh | Aligarh | GEN |
| West Bengal | Alipurduars | ST |
| Uttar Pradesh | Allahabad | GEN |
| Uttarakhand | Almora | SC |
| Rajasthan | Alwar | GEN |
| Andhra Pradesh | Amalapuram | SC |
| Haryana | Ambala | SC |
| Uttar Pradesh | Ambedkar Nagar | GEN |
| Uttar Pradesh | Amethi | GEN |
| Maharashtra | Amravati | SC |
| Gujarat | Amreli | GEN |
| Punjab | Amritsar | GEN |
| Uttar Pradesh | Amroha | GEN |
| Andhra Pradesh | Anakapalle | GEN |
| Gujarat | Anand | GEN |
| Punjab | Anandpur Sahib | GEN |
| Andhra Pradesh | Anantapur | GEN |
| Jammu & Kashmir | Anantnag | GEN |
| Andaman & Nicobar Islands | Andaman & Nicobar | GEN |
| Uttar Pradesh | Aonla | GEN |
| Tamil Nadu | Arakkonam | GEN |
| Andhra Pradesh | Araku | ST |
| West Bengal | Arambag | SC |
| Tamil Nadu | Arani | GEN |
| Bihar | Araria | GEN |
| Bihar | Arrah | GEN |
| Arunachal Pradesh | Arunachal East | GEN |
| Arunachal Pradesh | Arunachal West | GEN |
| West Bengal | Asansol | GEN |
| Odisha | Aska | GEN |
| Kerala | Attingal | GEN |
| Bihar | Aurangabad | GEN |
| Maharashtra | Aurangabad | GEN |
| Assam | Autonomous District | ST |
| Uttar Pradesh | Azamgarh | GEN |
| Uttar Pradesh | Badaun | GEN |
| Karnataka | Bagalkot | GEN |
| Uttar Pradesh | Baghpat | GEN |
| West Bengal | Baharampur | GEN |
| Uttar Pradesh | Bahraich | SC |
| Madhya Pradesh | Balaghat | GEN |
| Odisha | Balasore | GEN |
| Uttar Pradesh | Ballia | GEN |
| West Bengal | Balurghat | GEN |
| Gujarat | Banaskantha | GEN |
| Uttar Pradesh | Banda | GEN |
| Karnataka | Bangalore Central | GEN |
| Karnataka | Bangalore North | GEN |
| Karnataka | Bangalore Rural | GEN |
| Karnataka | Bangalore South | GEN |
| West Bengal | Bangaon | SC |
| Bihar | Banka | GEN |
| West Bengal | Bankura | GEN |
| Uttar Pradesh | Bansgaon | SC |
| Rajasthan | Banswara | ST |
| Andhra Pradesh | Bapatla | SC |
| Uttar Pradesh | Barabanki | SC |
| Maharashtra | Baramati | GEN |
| Jammu & Kashmir | Baramulla | GEN |
| West Bengal | Barasat | GEN |
| West Bengal | Bardhaman Purba | SC |
| West Bengal | Bardhaman-Durgapur | GEN |
| Gujarat | Bardoli | ST |
| Uttar Pradesh | Bareilly | GEN |
| Odisha | Bargarh | GEN |
| Rajasthan | Barmer | GEN |
| Assam | Barpeta | GEN |
| West Bengal | Barrackpur | GEN |
| West Bengal | Basirhat | GEN |
| Chhattisgarh | Bastar | ST |
| Uttar Pradesh | Basti | GEN |
| Punjab | Bathinda | GEN |
| Maharashtra | Beed | GEN |
| Bihar | Begusarai | GEN |
| Karnataka | Belgaum | GEN |
| Karnataka | Bellary | ST |
| Odisha | Berhampur | GEN |
| Madhya Pradesh | Betul | ST |
| Uttar Pradesh | Bhadohi | GEN |
| Odisha | Bhadrak | SC |
| Bihar | Bhagalpur | GEN |
| Maharashtra | Bhandara-Gondiya | GEN |
| Rajasthan | Bharatpur | SC |
| Gujarat | Bharuch | GEN |
| Gujarat | Bhavnagar | GEN |
| Rajasthan | Bhilwara | GEN |
| Madhya Pradesh | Bhind | SC |
| Maharashtra | Bhiwandi | GEN |
| Haryana | Bhiwani-Mahendragarh | GEN |
| Telangana | Bhongir | GEN |
| Madhya Pradesh | Bhopal | GEN |
| Odisha | Bhubaneswar | GEN |
| Karnataka | Bidar | GEN |
| Karnataka | Bijapur | SC |
| Uttar Pradesh | Bijnor | GEN |
| Rajasthan | Bikaner | SC |
| Chhattisgarh | Bilaspur | GEN |
| West Bengal | Birbhum | GEN |
| West Bengal | Bishnupur | SC |
| Odisha | Bolangir | GEN |
| West Bengal | Bolpur | SC |
| Uttar Pradesh | Bulandshahr | SC |
| Maharashtra | Buldhana | GEN |
| Bihar | Buxar | GEN |
| Kerala | Chalakudy | GEN |
| Karnataka | Chamarajanagar | SC |
| Uttar Pradesh | Chandauli | GEN |
| Chandigarh | Chandigarh | GEN |
| Nct Of Delhi | Chandni Chowk | GEN |
| Maharashtra | Chandrapur | GEN |
| Jharkhand | Chatra | GEN |
| Tamil Nadu | Chennai Central | GEN |
| Tamil Nadu | Chennai North | GEN |
| Tamil Nadu | Chennai South | GEN |
| Telangana | Chevella | GEN |
| Madhya Pradesh | Chhindwara | GEN |
| Gujarat | Chhota Udaipur | ST |
| Tamil Nadu | Chidambaram | SC |
| Karnataka | Chikkballapur | GEN |
| Karnataka | Chikkodi | GEN |
| Karnataka | Chitradurga | SC |
| Andhra Pradesh | Chittoor | SC |
| Rajasthan | Chittorgarh | GEN |
| Rajasthan | Churu | GEN |
| Tamil Nadu | Coimbatore | GEN |
| West Bengal | Coochbehar | SC |
| Tamil Nadu | Cuddalore | GEN |
| Odisha | Cuttack | GEN |
| Dadra & Nagar Haveli And Daman & Diu | Dadra & Nagar Haveli | ST |
| Gujarat | Dahod | ST |
| Karnataka | Dakshina Kannada | GEN |
| Dadra & Nagar Haveli And Daman & Diu | Daman & Diu | GEN |
| Madhya Pradesh | Damoh | GEN |
| Bihar | Darbhanga | GEN |
| West Bengal | Darjeeling | GEN |
| Rajasthan | Dausa | ST |
| Karnataka | Davanagere | GEN |
| Uttar Pradesh | Deoria | GEN |
| Madhya Pradesh | Dewas | SC |
| Jharkhand | Dhanbad | GEN |
| Madhya Pradesh | Dhar | ST |
| Tamil Nadu | Dharmapuri | GEN |
| Karnataka | Dharwad | GEN |
| Uttar Pradesh | Dhaurahra | GEN |
| Odisha | Dhenkanal | GEN |
| Assam | Dhubri | GEN |
| Maharashtra | Dhule | GEN |
| West Bengal | Diamond Harbour | GEN |
| Assam | Dibrugarh | GEN |
| Tamil Nadu | Dindigul | GEN |
| Maharashtra | Dindori | ST |
| Uttar Pradesh | Domariyaganj | GEN |
| West Bengal | Dum Dum | GEN |
| Jharkhand | Dumka | ST |
| Chhattisgarh | Durg | GEN |
| Nct Of Delhi | East Delhi | GEN |
| Andhra Pradesh | Eluru | GEN |
| Kerala | Ernakulam | GEN |
| Tamil Nadu | Erode | GEN |
| Uttar Pradesh | Etah | GEN |
| Uttar Pradesh | Etawah | SC |
| Uttar Pradesh | Faizabad | GEN |
| Haryana | Faridabad | GEN |
| Punjab | Faridkot | SC |
| Uttar Pradesh | Farrukhabad | GEN |
| Punjab | Fatehgarh Sahib | SC |
| Uttar Pradesh | Fatehpur Sikri | GEN |
| Uttar Pradesh | Fatehpur | GEN |
| Uttar Pradesh | Firozabad | GEN |
| Punjab | Firozpur | GEN |
| Maharashtra | Gadchiroli-Chimur | ST |
| Gujarat | Gandhinagar | GEN |
| Rajasthan | Ganganagar | SC |
| Uttarakhand | Garhwal | GEN |
| Assam | Gauhati | GEN |
| Uttar Pradesh | Gautam Buddha Nagar | GEN |
| Bihar | Gaya | SC |
| West Bengal | Ghatal | GEN |
| Uttar Pradesh | Ghaziabad | GEN |
| Uttar Pradesh | Ghazipur | GEN |
| Uttar Pradesh | Ghosi | GEN |
| Jharkhand | Giridih | GEN |
| Jharkhand | Godda | GEN |
| Uttar Pradesh | Gonda | GEN |
| Bihar | Gopalganj | SC |
| Uttar Pradesh | Gorakhpur | GEN |
| Karnataka | Gulbarga | SC |
| Madhya Pradesh | Guna | GEN |
| Andhra Pradesh | Guntur | GEN |
| Punjab | Gurdaspur | GEN |
| Haryana | Gurgaon | GEN |
| Madhya Pradesh | Gwalior | GEN |
| Bihar | Hajipur | SC |
| Himachal Pradesh | Hamirpur | GEN |
| Uttar Pradesh | Hamirpur | GEN |
| Uttar Pradesh | Hardoi | SC |
| Uttarakhand | Hardwar | GEN |
| Karnataka | Hassan | GEN |
| Uttar Pradesh | Hathras | SC |
| Maharashtra | Hatkanangle | GEN |
| Karnataka | Haveri | GEN |
| Jharkhand | Hazaribagh | GEN |
| Andhra Pradesh | Hindupur | GEN |
| Maharashtra | Hingoli | GEN |
| Haryana | Hisar | GEN |
| West Bengal | Hooghly | GEN |
| Madhya Pradesh | Hoshangabad | GEN |
| Punjab | Hoshiarpur | SC |
| West Bengal | Howrah | GEN |
| Telangana | Hyderabad | GEN |
| Kerala | Idukki | GEN |
| Madhya Pradesh | Indore | GEN |
| Manipur | Inner Manipur | GEN |
| Madhya Pradesh | Jabalpur | GEN |
| West Bengal | Jadavpur | GEN |
| Odisha | Jagatsinghpur | SC |
| Bihar | Jahanabad | GEN |
| Rajasthan | Jaipur Rural | GEN |
| Rajasthan | Jaipur | GEN |
| Odisha | Jajpur | SC |
| Punjab | Jalandhar | SC |
| Uttar Pradesh | Jalaun | SC |
| Maharashtra | Jalgaon | GEN |
| Maharashtra | Jalna | GEN |
| Rajasthan | Jalore | GEN |
| West Bengal | Jalpaiguri | SC |
| Jammu & Kashmir | Jammu | GEN |
| Gujarat | Jamnagar | GEN |
| Jharkhand | Jamshedpur | GEN |
| Bihar | Jamui | SC |
| West Bengal | Jangipur | GEN |
| Chhattisgarh | Janjgir-Champa | SC |
| Uttar Pradesh | Jaunpur | GEN |
| West Bengal | Jaynagar | SC |
| Rajasthan | Jhalawar-Baran | GEN |
| Bihar | Jhanjharpur | GEN |
| Uttar Pradesh | Jhansi | GEN |
| West Bengal | Jhargram | ST |
| Rajasthan | Jhunjhunu | GEN |
| Rajasthan | Jodhpur | GEN |
| Assam | Jorhat | GEN |
| Gujarat | Junagadh | GEN |
| Gujarat | Kachchh | SC |
| Andhra Pradesh | Kadapa | GEN |
| Uttar Pradesh | Kairana | GEN |
| Uttar Pradesh | Kaiserganj | GEN |
| Andhra Pradesh | Kakinada | GEN |
| Odisha | Kalahandi | GEN |
| Assam | Kaliabor | GEN |
| Tamil Nadu | Kallakurichi | GEN |
| Maharashtra | Kalyan | GEN |
| Tamil Nadu | Kancheepuram | SC |
| Odisha | Kandhamal | GEN |
| Himachal Pradesh | Kangra | GEN |
| Chhattisgarh | Kanker | ST |
| Uttar Pradesh | Kannauj | GEN |
| Tamil Nadu | Kanniyakumari | GEN |
| Kerala | Kannur | GEN |
| Uttar Pradesh | Kanpur | GEN |
| West Bengal | Kanthi | GEN |
| Bihar | Karakat | GEN |
| Rajasthan | Karauli -Dholpur | SC |
| Assam | Karimganj | SC |
| Telangana | Karimnagar | GEN |
| Haryana | Karnal | GEN |
| Tamil Nadu | Karur | GEN |
| Kerala | Kasaragod | GEN |
| Bihar | Katihar | GEN |
| Uttar Pradesh | Kaushambi | SC |
| Odisha | Kendrapara | GEN |
| Odisha | Keonjhar | ST |
| Punjab | Khadoor Sahib | GEN |
| Bihar | Khagaria | GEN |
| Madhya Pradesh | Khajuraho | GEN |
| Telangana | Khammam | GEN |
| Madhya Pradesh | Khandwa | GEN |
| Madhya Pradesh | Khargone | ST |
| Gujarat | Kheda | GEN |
| Uttar Pradesh | Kheri | GEN |
| Jharkhand | Khunti | ST |
| Bihar | Kishanganj | GEN |
| Jharkhand | Kodarma | GEN |
| Assam | Kokrajhar | ST |
| Karnataka | Kolar | SC |
| Maharashtra | Kolhapur | GEN |
| West Bengal | Kolkata Dakshin | GEN |
| West Bengal | Kolkata Uttar | GEN |
| Kerala | Kollam | GEN |
| Karnataka | Koppal | GEN |
| Odisha | Koraput | ST |
| Chhattisgarh | Korba | GEN |
| Rajasthan | Kota | GEN |
| Kerala | Kottayam | GEN |
| Kerala | Kozhikode | GEN |
| Tamil Nadu | Krishnagiri | GEN |
| West Bengal | Krishnanagar | GEN |
| Andhra Pradesh | Kurnool | GEN |
| Haryana | Kurukshetra | GEN |
| Uttar Pradesh | Kushi Nagar | GEN |
| Ladakh | Ladakh | GEN |
| Assam | Lakhimpur | GEN |
| Lakshadweep | Lakshadweep | ST |
| Uttar Pradesh | Lalganj | SC |
| Maharashtra | Latur | SC |
| Jharkhand | Lohardaga | ST |
| Uttar Pradesh | Lucknow | GEN |
| Punjab | Ludhiana | GEN |
| Uttar Pradesh | Machhlishahr | SC |
| Andhra Pradesh | Machilipatnam | GEN |
| Maharashtra | Madha | GEN |
| Bihar | Madhepura | GEN |
| Bihar | Madhubani | GEN |
| Tamil Nadu | Madurai | GEN |
| Telangana | Mahabubabad | ST |
| Bihar | Maharajganj | GEN |
| Uttar Pradesh | Maharajganj | GEN |
| Chhattisgarh | Mahasamund | GEN |
| Telangana | Mahbubnagar | GEN |
| Gujarat | Mahesana | GEN |
| Uttar Pradesh | Mainpuri | GEN |
| Kerala | Malappuram | GEN |
| West Bengal | Maldaha Dakshin | GEN |
| West Bengal | Maldaha Uttar | GEN |
| Telangana | Malkajgiri | GEN |
| Himachal Pradesh | Mandi | GEN |
| Madhya Pradesh | Mandla | ST |
| Madhya Pradesh | Mandsour | GEN |
| Karnataka | Mandya | GEN |
| Assam | Mangaldoi | GEN |
| Uttar Pradesh | Mathura | GEN |
| West Bengal | Mathurapur | SC |
| Maharashtra | Maval | GEN |
| Kerala | Mavelikkara | SC |
| Tamil Nadu | Mayiladuthurai | GEN |
| Odisha | Mayurbhanj | ST |
| Telangana | Medak | GEN |
| West Bengal | Medinipur | GEN |
| Uttar Pradesh | Meerut | GEN |
| Uttar Pradesh | Mirzapur | GEN |
| Uttar Pradesh | Misrikh | SC |
| Mizoram | Mizoram | ST |
| Uttar Pradesh | Mohanlalganj | SC |
| Uttar Pradesh | Moradabad | GEN |
| Madhya Pradesh | Morena | GEN |
| Maharashtra | Mumbai North-Central | GEN |
| Maharashtra | Mumbai North-East | GEN |
| Maharashtra | Mumbai North-West | GEN |
| Maharashtra | Mumbai North | GEN |
| Maharashtra | Mumbai South -Central | GEN |
| Maharashtra | Mumbai South | GEN |
| Bihar | Munger | GEN |
| West Bengal | Murshidabad | GEN |
| Uttar Pradesh | Muzaffarnagar | GEN |
| Bihar | Muzaffarpur | GEN |
| Karnataka | Mysore | GEN |
| Odisha | Nabarangpur | ST |
| Nagaland | Nagaland | GEN |
| Tamil Nadu | Nagapattinam | SC |
| Telangana | Nagarkurnool | SC |
| Rajasthan | Nagaur | GEN |
| Uttar Pradesh | Nagina | SC |
| Maharashtra | Nagpur | GEN |
| Uttarakhand | Nainital-Udhamsingh Nagar | GEN |
| Bihar | Nalanda | GEN |
| Telangana | Nalgonda | GEN |
| Tamil Nadu | Namakkal | GEN |
| Maharashtra | Nanded | GEN |
| Maharashtra | Nandurbar | ST |
| Andhra Pradesh | Nandyal | GEN |
| Andhra Pradesh | Narasaraopet | GEN |
| Andhra Pradesh | Narsapuram | GEN |
| Maharashtra | Nashik | GEN |
| Gujarat | Navsari | GEN |
| Bihar | Nawada | GEN |
| Andhra Pradesh | Nellore | GEN |
| Nct Of Delhi | New Delhi | GEN |
| Tamil Nadu | Nilgiris | SC |
| Telangana | Nizamabad | GEN |
| Nct Of Delhi | North East Delhi | GEN |
| Goa | North Goa | GEN |
| Nct Of Delhi | North West Delhi | SC |
| Assam | Nowgong | GEN |
| Andhra Pradesh | Ongole | GEN |
| Maharashtra | Osmanabad | GEN |
| Manipur | Outer Manipur | ST |
| Kerala | Palakkad | GEN |
| Jharkhand | Palamu | SC |
| Maharashtra | Palghar | ST |
| Rajasthan | Pali | GEN |
| Gujarat | Panchmahal | GEN |
| Maharashtra | Parbhani | GEN |
| Bihar | Paschim Champaran | GEN |
| Bihar | Pataliputra | GEN |
| Gujarat | Patan | GEN |
| Kerala | Pathanamthitta | GEN |
| Punjab | Patiala | GEN |
| Bihar | Patna Sahib | GEN |
| Telangana | Peddapalle | SC |
| Tamil Nadu | Perambalur | GEN |
| Uttar Pradesh | Phulpur | GEN |
| Uttar Pradesh | Pilibhit | GEN |
| Tamil Nadu | Pollachi | GEN |
| Puducherry | Pondicherry | GEN |
| Kerala | Ponnani | GEN |
| Gujarat | Porbandar | GEN |
| Uttar Pradesh | Pratapgarh | GEN |
| Maharashtra | Pune | GEN |
| Odisha | Puri | GEN |
| Bihar | Purnia | GEN |
| West Bengal | Purulia | GEN |
| Bihar | Purvi Champaran | GEN |
| Uttar Pradesh | Rae Bareli | GEN |
| Karnataka | Raichur | ST |
| Maharashtra | Raigad | GEN |
| West Bengal | Raiganj | GEN |
| Chhattisgarh | Raigarh | ST |
| Chhattisgarh | Raipur | GEN |
| Andhra Pradesh | Rajahmundry | GEN |
| Andhra Pradesh | Rajampet | GEN |
| Madhya Pradesh | Rajgarh | GEN |
| Gujarat | Rajkot | GEN |
| Jharkhand | Rajmahal | ST |
| Chhattisgarh | Rajnandgaon | GEN |
| Rajasthan | Rajsamand | GEN |
| Tamil Nadu | Ramanathapuram | GEN |
| Uttar Pradesh | Rampur | GEN |
| Maharashtra | Ramtek | SC |
| West Bengal | Ranaghat | SC |
| Jharkhand | Ranchi | GEN |
| Madhya Pradesh | Ratlam | ST |
| Maharashtra | Ratnagiri -Sindhudurg | GEN |
| Maharashtra | Raver | GEN |
| Madhya Pradesh | Rewa | GEN |
| Uttar Pradesh | Robertsganj | SC |
| Haryana | Rohtak | GEN |
| Gujarat | Sabarkantha | GEN |
| Madhya Pradesh | Sagar | GEN |
| Uttar Pradesh | Saharanpur | GEN |
| Tamil Nadu | Salem | GEN |
| Uttar Pradesh | Salempur | GEN |
| Bihar | Samastipur | SC |
| Odisha | Sambalpur | GEN |
| Uttar Pradesh | Sambhal | GEN |
| Maharashtra | Sangli | GEN |
| Punjab | Sangrur | GEN |
| Uttar Pradesh | Sant Kabir Nagar | GEN |
| Bihar | Saran | GEN |
| Bihar | Sasaram | SC |
| Maharashtra | Satara | GEN |
| Madhya Pradesh | Satna | GEN |
| Telangana | Secunderabad | GEN |
| Madhya Pradesh | Shahdol | ST |
| Uttar Pradesh | Shahjahanpur | SC |
| Bihar | Sheohar | GEN |
| Meghalaya | Shillong | ST |
| Himachal Pradesh | Shimla | SC |
| Karnataka | Shimoga | GEN |
| Maharashtra | Shirdi | SC |
| Maharashtra | Shirur | GEN |
| Uttar Pradesh | Shrawasti | GEN |
| Madhya Pradesh | Sidhi | GEN |
| Rajasthan | Sikar | GEN |
| Sikkim | Sikkim | GEN |
| Assam | Silchar | GEN |
| Jharkhand | Singhbhum | ST |
| Haryana | Sirsa | SC |
| Bihar | Sitamarhi | GEN |
| Uttar Pradesh | Sitapur | GEN |
| Tamil Nadu | Sivaganga | GEN |
| Bihar | Siwan | GEN |
| Maharashtra | Solapur | SC |
| Haryana | Sonipat | GEN |
| Nct Of Delhi | South Delhi | GEN |
| Goa | South Goa | GEN |
| West Bengal | Sreerampur | GEN |
| Andhra Pradesh | Srikakulam | GEN |
| Jammu & Kashmir | Srinagar | GEN |
| Tamil Nadu | Sriperumbudur | GEN |
| Uttar Pradesh | Sultanpur | GEN |
| Odisha | Sundargarh | ST |
| Bihar | Supaul | GEN |
| Gujarat | Surat | GEN |
| Gujarat | Surendranagar | GEN |
| Chhattisgarh | Surguja | ST |
| West Bengal | Tamluk | GEN |
| Uttarakhand | Tehri Garhwal | GEN |
| Tamil Nadu | Tenkasi | SC |
| Assam | Tezpur | GEN |
| Maharashtra | Thane | GEN |
| Tamil Nadu | Thanjavur | GEN |
| Tamil Nadu | Theni | GEN |
| Kerala | Thiruvananthapuram | GEN |
| Tamil Nadu | Thoothukkudi | GEN |
| Kerala | Thrissur | GEN |
| Madhya Pradesh | Tikamgarh | SC |
| Tamil Nadu | Tiruchirappalli | GEN |
| Tamil Nadu | Tirunelveli | GEN |
| Andhra Pradesh | Tirupati | SC |
| Tamil Nadu | Tiruppur | GEN |
| Tamil Nadu | Tiruvallur | SC |
| Tamil Nadu | Tiruvannamalai | GEN |
| Rajasthan | Tonk - Sawai Madhopur | GEN |
| Tripura | Tripura East | ST |
| Tripura | Tripura West | GEN |
| Karnataka | Tumkur | GEN |
| Meghalaya | Tura | ST |
| Rajasthan | Udaipur | ST |
| Jammu & Kashmir | Udhampur | GEN |
| Karnataka | Udupi Chikmagalur | GEN |
| Bihar | Ujiarpur | GEN |
| Madhya Pradesh | Ujjain | SC |
| West Bengal | Uluberia | GEN |
| Uttar Pradesh | Unnao | GEN |
| Karnataka | Uttara Kannada | GEN |
| Kerala | Vadakara | GEN |
| Gujarat | Vadodara | GEN |
| Bihar | Vaishali | GEN |
| Bihar | Valmiki Nagar | GEN |
| Gujarat | Valsad | ST |
| Uttar Pradesh | Varanasi | GEN |
| Tamil Nadu | Vellore | GEN |
| Madhya Pradesh | Vidisha | GEN |
| Andhra Pradesh | Vijayawada | GEN |
| Tamil Nadu | Viluppuram | SC |
| Tamil Nadu | Virudhunagar | GEN |
| Andhra Pradesh | Visakhapatnam | GEN |
| Andhra Pradesh | Vizianagaram | GEN |
| Telangana | Warangal | SC |
| Maharashtra | Wardha | GEN |
| Kerala | Wayanad | GEN |
| Nct Of Delhi | West Delhi | GEN |
| Maharashtra | Yavatmal-Washim | GEN |
| Telangana | Zahirabad | GEN |

**Supplementary Table 4. Effective Sample Sizes for 122 Indicators, 2021 and 2016**

| **Indicator No** | **Category-Indicator Label** | **2021** | | | **2016** | | |
| --- | --- | --- | --- | --- | --- | --- | --- |
|  |  | **State ESS** | **District ESS** | **Cluster ESS** | **State ESS** | **District ESS** | **Cluster ESS** |
|  | **Socio-Economic Profile** |  |  |  |  |  |  |
| 1 | Population with BPL cards | 962 | 628 | 1266 | 754 | 405 | 651 |
|  | **Health Care** |  |  |  |  |  |  |
| 2 | Acute Respiratory Infection [All Children] | 1466 | 1144 | 588 | 1207 | 1355 | 779 |
| 3 | Acute Respiratory Infection [Children Getting Treatment - Facility] | 1901 | 605 | 590 | 2041 | 702 | 703 |
| 4 | Diarrhoea [Received ORS] | 1564 | 359 | 647 | 1818 | 761 | 696 |
| 5 | Diarrhoea [Received Zinc] | 1551 | 565 | 533 | 1400 | 830 | 566 |
| 6 | Diarrhoea Treatment [Facility] | 2209 | 243 | 379 | 2220 | 677 | 487 |
| 7 | DPT Vaccination [3 Doses] | 2065 | 979 | 553 | 1762 | 1561 | 749 |
| 8 | Full Vaccination | 1808 | 1344 | 600 | 1812 | 1510 | 812 |
| 9 | Full Vaccination [Vaccination Card] | 1753 | 979 | 328 | 2264 | 1092 | 457 |
| 10 | Health Insurance [Any] | 1733 | 672 | 2066 | 624 | 212 | 591 |
| 11 | Hepatitis B Vaccine [3 Doses] | 1697 | 1036 | 436 | 1817 | 1523 | 662 |
| 12 | ICDS Benefits [Children] | 1542 | 1466 | 1890 | 1579 | 1327 | 2060 |
| 13 | Low Birth Weight | 2125 | 1588 | 903 | 2212 | 1500 | 799 |
| 14 | Measles-Containing Vaccine [First Dose] | 2320 | 1008 | 501 | 1969 | 1421 | 535 |
| 15 | Measles-Containing Vaccine [Second Dose] | 1929 | 1298 | 903 |  |  |  |
| 16 | Polio Vaccination [3 Doses] | 1839 | 1217 | 570 | 2216 | 1354 | 739 |
| 17 | Rotavirus Vaccine [3 Doses] | 2095 | 1105 | 565 |  |  |  |
| 18 | Vitamin A Dose | 1895 | 1260 | 1178 | 1699 | 1581 | 1350 |
| 19 | Zero Dose [Child Immunization] | 1937 | 625 | 363 | 1641 | 1060 | 560 |
|  | **Maternal Health and Family Planning** |  |  |  |  |  |  |
| 20 | Antenatal Care Visit [Four or More] | 2031 | 1536 | 1753 | 1937 | 1756 | 1533 |
| 21 | Antenatal Care Visit [First Trimester] | 1493 | 1634 | 1374 | 1712 | 1712 | 1814 |
| 22 | Birth Registration | 930 | 481 | 423 | 2740 | 1506 | 1433 |
| 23 | Birth Weight Recorded | 2217 | 1345 | 966 | 2236 | 1374 | 1506 |
| 24 | Caesarean Section Delivery | 1951 | 1733 | 1241 | 1493 | 1619 | 1181 |
| 25 | Caesarean Section in Private Sector | 1951 | 1243 | 1036 | 1846 | 1427 | 928 |
| 26 | Caesarean Section in Public Sector | 1950 | 1350 | 953 | 2108 | 1287 | 667 |
| 27 | Childbirths in Public Facility | 1572 | 1467 | 1711 | 1349 | 1623 | 2122 |
| 28 | Condom | 1921 | 1469 | 1138 | 1858 | 1779 | 1496 |
| 29 | Family Planning [Any Methods by Women] | 809 | 1808 | 1955 | 1171 | 1908 | 2353 |
| 30 | Family Planning [Modern] | 1320 | 1867 | 2010 | 832 | 1889 | 2039 |
| 31 | Family Planning [Unmet Need] | 1083 | 1620 | 1438 | 1462 | 2023 | 1203 |
| 32 | Family Planning Services Quality [Family Planning Counselling] | 1193 | 1241 | 2154 | 1189 | 1190 | 1806 |
| 33 | Female Sterilization | 2224 | 1534 | 1755 | 1880 | 1359 | 2151 |
| 34 | Family Planning Services Quality [Side Effects Counselling] | 1649 | 1264 | 967 | 1500 | 1354 | 1124 |
| 35 | Home Delivery by Skilled Health Personnel | 2105 | 880 | 544 | 1624 | 1202 | 767 |
| 36 | Injectables | 1377 | 911 | 605 | 2125 | 447 | 343 |
| 37 | Institutional Childbirth | 2268 | 1328 | 1021 | 2027 | 1443 | 1622 |
| 38 | Iron Folic Acid [100 days or More] | 1536 | 1627 | 1785 | 1872 | 1459 | 1477 |
| 39 | Iron Folic Acid [180 days or More] | 1638 | 1309 | 1601 | 1748 | 1359 | 787 |
| 40 | IUD/PPIUD | 1951 | 1531 | 926 | 1659 | 1605 | 708 |
| 41 | Male Sterilization | 1456 | 712 | 460 | 1225 | 756 | 520 |
| 42 | Maternal Care Quality [Postpartum] | 1694 | 824 | 251 | 1760 | 1282 | 761 |
| 43 | Mother and Child Protection Card | 1441 | 1046 | 624 | 1844 | 1589 | 969 |
| 44 | Neonatal Tetanus | 1444 | 1102 | 823 | 1406 | 1615 | 1007 |
| 45 | Pill | 2159 | 1572 | 1182 | 2494 | 1376 | 1156 |
| 46 | Postnatal Care [Mothers] | 1930 | 1699 | 1270 | 1278 | 1728 | 1871 |
| 47 | Pregnancy Registration | 1867 | 1500 | 765 | 1440 | 1739 | 1262 |
| 48 | Skilled Birth Attendance | 1948 | 1332 | 920 | 2303 | 1315 | 1408 |
| 49 | Unmet Need for Spacing | 1621 | 2191 | 828 | 1756 | 1630 | 908 |
|  | **Morbidity and Mortality** |  |  |  |  |  |  |
| 50 | Diarrhoea [Children] | 1299 | 1498 | 845 | 1562 | 1419 | 916 |
| 51 | Elevated Blood Pressure or On Medication [Men] | 374 | 310 | 568 | 1030 | 360 | 401 |
| 52 | Elevated Blood Pressure or On Medication [Women] | 341 | 322 | 326 | 500 | 687 | 578 |
| 53 | High Blood Sugar [Men] | 496 | 251 | 157 | 1368 | 292 | 278 |
| 54 | High Blood Sugar [Women] | 784 | 685 | 338 | 1282 | 1633 | 820 |
| 55 | High or Very High Blood Sugar or On Medication [Men] | 304 | 325 | 261 | 1788 | 587 | 536 |
| 56 | High or Very High Blood Sugar or On Medication [Women] | 706 | 755 | 418 | 1251 | 1848 | 1074 |
| 57 | Mildly Elevated Blood Pressure [Men] | 794 | 608 | 598 | 1818 | 853 | 777 |
| 58 | Mildly Elevated Blood Pressure [Women] | 716 | 575 | 631 | 1624 | 1615 | 781 |
| 59 | Moderate or Severe Blood Pressure [Men] | 524 | 663 | 306 | 1937 | 164 | 197 |
| 60 | Moderate or Severe Blood Pressure [Women] | 672 | 521 | 308 | 1615 | 1432 | 533 |
| 61 | Probability of Dying before Five Years | 2128 | 673 | 513 | 2627 | 655 | 690 |
| 62 | Probability of Dying before One Year | 2244 | 697 | 468 | 1998 | 539 | 675 |
| 63 | Probability of Dying within 28 Days | 1977 | 464 | 475 | 2012 | 288 | 645 |
| 64 | Risky Waist-to-hip Ratio [Women] | 1402 | 1117 | 2549 |  |  |  |
| 65 | Very High Blood Sugar [Men] | 769 | 661 | 289 | 2021 | 174 | 392 |
| 66 | Very High Blood Sugar [Women] | 1794 | 1285 | 392 | 2072 | 1514 | 415 |
|  | **Nutrition [Clinical/Anthropometry]** |  |  |  |  |  |  |
| 67 | Anaemia [Any - Adolescent Women] | 2079 | 1557 | 1078 | 1334 | 1742 | 951 |
| 68 | Anaemia [Any - All Women] | 1187 | 1641 | 1965 | 697 | 1606 | 2214 |
| 69 | Anaemia [Any - Pregnant Women] | 1821 | 833 | 413 | 1691 | 1276 | 306 |
| 70 | Child Anaemia [Any] | 1739 | 1597 | 1913 | 1208 | 1740 | 1707 |
| 71 | Child Stunting | 1713 | 1540 | 1577 | 1658 | 1619 | 1281 |
| 72 | Child Underweight | 2162 | 1491 | 1468 | 1950 | 1521 | 1262 |
| 73 | Child Wasting | 1743 | 1392 | 1112 | 1580 | 1641 | 1223 |
| 74 | Mild Anaemia [Children] | 1943 | 1333 | 665 | 2042 | 1648 | 385 |
| 75 | Mild Anaemia [Women] | 1876 | 1539 | 925 | 1355 | 1518 | 1731 |
| 76 | Moderate Anaemia [Children] | 1912 | 1819 | 1430 | 1370 | 1802 | 1314 |
| 77 | Moderate Anaemia [Women] | 1673 | 1829 | 1748 | 1069 | 1817 | 2142 |
| 78 | Overweight Children | 1501 | 1057 | 674 | 1444 | 1147 | 450 |
| 79 | Overweight or Obese [Women] | 1273 | 1932 | 2285 | 1046 | 1603 | 1891 |
| 80 | Severe Anaemia [Children] | 2146 | 701 | 494 | 2033 | 764 | 497 |
| 81 | Severe Anaemia [Women] | 1039 | 1347 | 1092 | 1560 | 1651 | 924 |
| 82 | Severe Stunting [Children] | 1468 | 1478 | 895 | 1685 | 1490 | 1186 |
| 83 | Severe Underweight [Children] | 2373 | 1262 | 1050 | 2382 | 1525 | 727 |
| 84 | Severe Wasting [Children] | 1559 | 1416 | 914 | 1220 | 1644 | 1088 |
| 85 | Underweight [Women] | 1866 | 1626 | 1963 | 2047 | 2118 | 1730 |
|  | **Nutrition [Diet]** |  |  |  |  |  |  |
| 86 | Adequate Diet [Breastfed Children] | 2321 | 614 | 258 | 2097 | 793 | 387 |
| 87 | Adequate Diet [Non-breastfed Children] | 1404 | 4 | 117 | 1557 | 14 | 153 |
| 88 | Adequate Diet [Total] | 2053 | 695 | 448 | 2032 | 841 | 421 |
| 89 | Early Breastfeeding Initiation | 1592 | 1326 | 1457 | 1784 | 1096 | 1611 |
| 90 | ICDS Supplementary Nutrition | 1696 | 1432 | 1620 | 1934 | 1468 | 2093 |
| 91 | Iodized Salt Intake | 1223 | 236 | 283 | 368 | 224 | 211 |
| 92 | Exclusive Breastfeeding [Under 6 Months] | 2034 | 400 | 360 | 1544 | 1130 | 129 |
| 93 | Receiving Solid/Semi-solid Food [6-8 Months] | 1794 | 290 | 122 | 1654 | 341 | 59 |
| 94 | Zero Food [Children] | 2114 | 1260 | 635 | 1762 | 1453 | 740 |
|  | **Social Infrastructure** |  |  |  |  |  |  |
| 95 | Access to Electricity | 1303 | 542 | 450 | 1222 | 16 | 710 |
| 96 | Clean Cooking Fuel | 388 | 393 | 627 | 110 | 70 | 1454 |
| 97 | Death Registration | 2207 | 1617 | 846 |  |  |  |
| 98 | Handwashing Facilities | 697 | 322 | 729 | 224 | 259 | 665 |
| 99 | Hygienic Protection Methods [Menstruation] | 1692 | 1401 | 1690 | 1466 | 1253 | 1794 |
| 100 | Improved Sanitation Facility | 223 | 119 | 398 | 487 | 311 | 766 |
| 101 | Improved Source of Drinking Water | 72 | 13 | 81 | 413 | 15 | 275 |
| 102 | Internet Usage [Women] | 1393 | 1034 | 1967 |  |  |  |
| 103 | Private Latrine | 777 | 571 | 1196 | 245 | 111 | 858 |
| 104 | Safe Stool Disposal | 1736 | 1412 | 1140 | 1644 | 1326 | 1440 |
| 105 | Women with Personal Mobile Phone | 1647 | 1071 | 2084 | 1626 | 1043 | 1782 |
| 106 | Alcohol Consumption [Men] | 1138 | 838 | 1194 | 1599 | 1200 | 1898 |
| 107 | Alcohol Consumption [Women] | 1513 | 1354 | 705 | 1741 | 1079 | 711 |
| 108 | Child Marriage [Boy] | 2148 | 431 | 403 | 1937 | 579 | 421 |
| 109 | Child Marriage [Girl] | 2225 | 1940 | 820 | 1764 | 1835 | 1063 |
| 110 | Currently Working Women | 2056 | 1024 | 1503 | 1799 | 1049 | 1755 |
| 111 | Female School Attendance | 292 | 284 | 449 | 639 | 610 | 752 |
| 112 | High School Matriculation [Men] | 1674 | 941 | 1632 | 1612 | 940 | 2046 |
| 113 | High School Matriculation [Women] | 1428 | 1460 | 2392 | 1488 | 1408 | 2310 |
| 114 | Intimate Partner Violence [Against Women] | 1830 | 830 | 1341 | 2190 | 839 | 1392 |
| 115 | Literacy [Men] | 1783 | 953 | 1584 | 2197 | 1027 | 1564 |
| 116 | Literacy [Women] | 1484 | 1456 | 2542 | 1425 | 1682 | 2436 |
| 117 | Population below 15 Years | 254 | 330 | 491 | 488 | 664 | 605 |
| 118 | Sexual Violence [Young Women] | 1032 | 28 | 170 | 1723 | 21 | 169 |
| 119 | Teenage Pregnancy | 1842 | 1325 | 417 | 1991 | 1459 | 542 |
| 120 | Tobacco Consumption [Women] | 1922 | 1270 | 1260 | 1643 | 1447 | 1694 |
| 121 | Tobacco Use [Men] | 415 | 302 | 638 | 1793 | 1044 | 1639 |
| 122 | Women Participation in Household Decisions | 1781 | 603 | 964 | 2081 | 822 | 1288 |

*Note*. The following indicators were excluded from 2016 estimates due to data limitations: Measles-Containing Vaccine (2nd Dose), Rotavirus Vaccine (3 Doses), Risky Waist-to-Hip Ratio (Women), Death Registration, Internet Usage (Women).

**Supplementary Table 5. Multilevel Model Estimates for 122 Indicators, 2021 and 2016**

| **Indicator No** | **Category-Indicator Label** | **Fixed Part, 2021** | **Random Part, 2021** | | | **Fixed Part, 2016** | **Random Part, 2016** | | |
| --- | --- | --- | --- | --- | --- | --- | --- | --- | --- |
|  |  | **Estimate (SE)** | **State Variance (SE)** | **Distrtict Variance (SE)** | **Cluster Variance (SE)** | **Estimate (SE)** | **State Variance (SE)** | **Distrtict Variance (SE)** | **Cluster Variance (SE)** |
|  | **Socio-Economic Profile** |  |  |  |  |  |  |  |  |
| 1 | Population with BPL cards | -0.2 (0.3) | 2.3 (0.6) | 0.4 (0.0) | 1.4 (0.0) | -1.0 (0.3) | 2.3 (0.6) | 0.4 (0.0) | 1.5 (0.0) |
|  | **Health Care** |  |  |  |  |  |  |  |  |
| 2 | Acute Respiratory Infection [All Children] | -4.7 (0.1) | 0.3 (0.1) | 0.4 (0.0) | 1.7 (0.1) | -4.5 (0.1) | 0.3 (0.1) | 0.5 (0.0) | 1.1 (0.0) |
| 3 | Acute Respiratory Infection [Children Getting Treatment - Facility] | 1.2 (0.1) | 0.6 (0.2) | 0.1 (0.0) | 0.9 (0.1) | 1.2 (0.2) | 0.9 (0.2) | 0.1 (0.0) | 1.0 (0.1) |
| 4 | Diarrhoea [Received ORS] | 0.7 (0.1) | 0.2 (0.1) | 0.2 (0.0) | 1.2 (0.1) | 0.5 (0.1) | 0.3 (0.1) | 0.2 (0.0) | 0.8 (0.1) |
| 5 | Diarrhoea [Received Zinc] | -1.0 (0.1) | 0.3 (0.1) | 0.3 (0.0) | 1.8 (0.2) | -1.6 (0.1) | 0.5 (0.2) | 0.4 (0.1) | 1.9 (0.2) |
| 6 | Diarrhoea Treatment [Facility] | 1.0 (0.1) | 0.4 (0.1) | 0.1 (0.0) | 0.7 (0.1) | 0.9 (0.1) | 0.7 (0.2) | 0.2 (0.0) | 0.8 (0.1) |
| 7 | DPT Vaccination [3 Doses] | 2.5 (0.1) | 0.4 (0.1) | 0.3 (0.0) | 0.9 (0.1) | 2.0 (0.2) | 0.8 (0.2) | 0.5 (0.0) | 1.1 (0.1) |
| 8 | Full Vaccination | 1.7 (0.1) | 0.3 (0.1) | 0.3 (0.0) | 0.8 (0.1) | 0.9 (0.1) | 0.6 (0.2) | 0.3 (0.0) | 0.8 (0.0) |
| 9 | Full Vaccination [Vaccination Card] | 2.2 (0.1) | 0.4 (0.1) | 0.3 (0.0) | 0.9 (0.1) | 1.7 (0.1) | 0.5 (0.2) | 0.3 (0.0) | 1.0 (0.1) |
| 10 | Health Insurance [Any] | -0.4 (0.3) | 2.3 (0.6) | 0.2 (0.0) | 1.2 (0.0) | -1.6 (0.3) | 3.3 (0.9) | 0.5 (0.0) | 2.0 (0.0) |
| 11 | Hepatitis B Vaccine [3 Doses] | 2.1 (0.1) | 0.5 (0.1) | 0.4 (0.0) | 1.1 (0.1) | 0.9 (0.1) | 0.7 (0.2) | 0.4 (0.0) | 0.9 (0.0) |
| 12 | ICDS Benefits [Children] | 0.9 (0.1) | 0.5 (0.2) | 0.3 (0.0) | 1.3 (0.0) | 0.2 (0.1) | 0.7 (0.2) | 0.4 (0.0) | 1.2 (0.0) |
| 13 | Low Birth Weight | -1.9 (0.1) | 0.2 (0.1) | 0.1 (0.0) | 0.3 (0.0) | -1.8 (0.1) | 0.2 (0.1) | 0.1 (0.0) | 0.3 (0.0) |
| 14 | Measles-Containing Vaccine [First Dose] | 2.6 (0.1) | 0.4 (0.1) | 0.3 (0.0) | 1.0 (0.1) | 2.1 (0.2) | 0.7 (0.2) | 0.4 (0.0) | 0.9 (0.1) |
| 15 | Measles-Containing Vaccine [Second Dose] | 0.4 (0.2) | 0.7 (0.2) | 0.3 (0.0) | 1.1 (0.1) |  |  |  |  |
| 16 | Polio Vaccination [3 Doses] | 1.9 (0.1) | 0.3 (0.1) | 0.3 (0.0) | 0.9 (0.1) | 1.4 (0.1) | 0.6 (0.2) | 0.3 (0.0) | 0.8 (0.0) |
| 17 | Rotavirus Vaccine [3 Doses] | -1.6 (0.5) | 7.3 (2.0) | 0.6 (0.1) | 1.4 (0.1) |  |  |  |  |
| 18 | Vitamin A Dose | 1.0 (0.1) | 0.7 (0.2) | 0.2 (0.0) | 1.2 (0.0) | 0.8 (0.1) | 0.7 (0.2) | 0.3 (0.0) | 1.0 (0.0) |
| 19 | Zero Dose [Child Immunization] | -3.4 (0.1) | 0.4 (0.1) | 0.3 (0.0) | 1.2 (0.1) | -3.1 (0.2) | 1.0 (0.3) | 0.6 (0.1) | 1.3 (0.1) |
|  | **Maternal Health and Family Planning** |  |  |  |  |  |  |  |  |
| 20 | Antenatal Care Visit [Four or More] | 1.1 (0.2) | 1.5 (0.4) | 0.4 (0.0) | 1.4 (0.0) | 0.7 (0.3) | 2.2 (0.6) | 0.5 (0.0) | 1.2 (0.0) |
| 21 | Antenatal Care Visit [First Trimester] | 1.4 (0.1) | 0.6 (0.2) | 0.3 (0.0) | 1.0 (0.0) | 0.8 (0.2) | 0.8 (0.2) | 0.4 (0.0) | 0.8 (0.0) |
| 22 | Birth Registration | 3.8 (0.2) | 1.7 (0.5) | 0.4 (0.0) | 1.3 (0.0) | 3.0 (0.3) | 2.5 (0.7) | 0.5 (0.0) | 1.4 (0.0) |
| 23 | Birth Weight Recorded | 3.9 (0.2) | 1.9 (0.5) | 0.5 (0.0) | 1.5 (0.0) | 2.8 (0.3) | 3.1 (0.8) | 0.7 (0.0) | 1.4 (0.0) |
| 24 | Caesarean Section Delivery | -1.4 (0.2) | 0.9 (0.2) | 0.3 (0.0) | 0.8 (0.0) | -1.9 (0.2) | 0.8 (0.2) | 0.4 (0.0) | 0.9 (0.0) |
| 25 | Caesarean Section in Private Sector | 0.1 (0.1) | 0.7 (0.2) | 0.2 (0.0) | 0.8 (0.0) | -0.2 (0.1) | 0.5 (0.1) | 0.2 (0.0) | 0.7 (0.0) |
| 26 | Caesarean Section in Public Sector | -1.9 (0.2) | 0.9 (0.2) | 0.3 (0.0) | 0.9 (0.0) | -2.2 (0.2) | 0.8 (0.2) | 0.3 (0.0) | 0.9 (0.0) |
| 27 | Childbirths in Public Facility | 0.9 (0.1) | 0.7 (0.2) | 0.3 (0.0) | 1.0 (0.0) | 0.4 (0.2) | 0.7 (0.2) | 0.3 (0.0) | 0.9 (0.0) |
| 28 | Condom | -2.9 (0.2) | 1.3 (0.4) | 0.2 (0.0) | 0.5 (0.0) | -3.7 (0.2) | 1.7 (0.4) | 0.4 (0.0) | 0.6 (0.0) |
| 29 | Family Planning [Any Methods by Women] | 0.7 (0.1) | 0.2 (0.1) | 0.2 (0.0) | 0.3 (0.0) | 0.0 (0.1) | 0.5 (0.1) | 0.3 (0.0) | 0.4 (0.0) |
| 30 | Family Planning [Modern] | 0.2 (0.1) | 0.3 (0.1) | 0.2 (0.0) | 0.3 (0.0) | -0.3 (0.1) | 0.5 (0.1) | 0.3 (0.0) | 0.3 (0.0) |
| 31 | Family Planning [Unmet Need] | -2.5 (0.1) | 0.1 (0.0) | 0.2 (0.0) | 0.3 (0.0) | -1.9 (0.1) | 0.3 (0.1) | 0.1 (0.0) | 0.2 (0.0) |
| 32 | Family Planning Services Quality [Family Planning Counselling] | -1.1 (0.1) | 0.2 (0.1) | 0.2 (0.0) | 0.7 (0.0) | -1.5 (0.1) | 0.3 (0.1) | 0.2 (0.0) | 0.8 (0.0) |
| 33 | Female Sterilization | -1.0 (0.2) | 1.1 (0.3) | 0.2 (0.0) | 0.3 (0.0) | -1.1 (0.2) | 1.1 (0.3) | 0.3 (0.0) | 0.4 (0.0) |
| 34 | Family Planning Services Quality [Side Effects Counselling] | 0.8 (0.1) | 0.6 (0.2) | 0.3 (0.0) | 1.6 (0.1) | 0.0 (0.1) | 0.6 (0.2) | 0.3 (0.0) | 1.5 (0.0) |
| 35 | Home Delivery by Skilled Health Personnel | -4.8 (0.2) | 1.3 (0.4) | 0.4 (0.0) | 1.6 (0.1) | -4.3 (0.2) | 0.9 (0.3) | 0.4 (0.0) | 1.5 (0.0) |
| 36 | Injectables | -6.4 (0.2) | 1.6 (0.5) | 0.5 (0.1) | 1.1 (0.1) | -7.5 (0.2) | 1.3 (0.4) | 0.4 (0.1) | 1.2 (0.2) |
| 37 | Institutional Childbirth | 3.5 (0.3) | 2.8 (0.8) | 0.6 (0.0) | 1.6 (0.0) | 2.5 (0.3) | 3.2 (0.9) | 0.7 (0.0) | 1.5 (0.0) |
| 38 | Iron Folic Acid [100 days or More] | 0.1 (0.2) | 1.5 (0.4) | 0.3 (0.0) | 1.0 (0.0) | -0.7 (0.2) | 1.5 (0.4) | 0.3 (0.0) | 1.0 (0.0) |
| 39 | Iron Folic Acid [180 days or More] | -1.2 (0.2) | 1.6 (0.4) | 0.3 (0.0) | 1.2 (0.0) | -2.3 (0.3) | 2.2 (0.6) | 0.5 (0.0) | 1.3 (0.0) |
| 40 | IUD/PPIUD | -4.1 (0.2) | 1.1 (0.3) | 0.2 (0.0) | 0.7 (0.0) | -4.5 (0.2) | 0.9 (0.2) | 0.4 (0.0) | 0.6 (0.0) |
| 41 | Male Sterilization | -7.7 (0.3) | 3.1 (1.0) | 1.8 (0.2) | 1.3 (0.1) | -8.1 (0.3) | 3.3 (1.2) | 1.6 (0.2) | 1.7 (0.1) |
| 42 | Maternal Care Quality [Postpartum] | 4.8 (0.2) | 1.1 (0.3) | 0.7 (0.1) | 2.4 (0.2) | 3.2 (0.2) | 1.3 (0.4) | 0.4 (0.0) | 1.7 (0.1) |
| 43 | Mother and Child Protection Card | 4.3 (0.2) | 0.9 (0.3) | 0.6 (0.1) | 1.7 (0.1) | 3.1 (0.2) | 1.3 (0.4) | 0.4 (0.0) | 1.2 (0.0) |
| 44 | Neonatal Tetanus | 2.9 (0.1) | 0.3 (0.1) | 0.3 (0.0) | 1.0 (0.0) | 2.8 (0.1) | 0.7 (0.2) | 0.4 (0.0) | 1.0 (0.0) |
| 45 | Pill | -3.8 (0.3) | 2.3 (0.6) | 0.3 (0.0) | 0.6 (0.0) | -4.2 (0.3) | 2.6 (0.7) | 0.3 (0.0) | 0.5 (0.0) |
| 46 | Postnatal Care [Mothers] | 1.8 (0.2) | 1.0 (0.3) | 0.3 (0.0) | 1.0 (0.0) | 0.9 (0.2) | 1.0 (0.3) | 0.4 (0.0) | 1.1 (0.0) |
| 47 | Pregnancy Registration | 3.9 (0.2) | 0.9 (0.2) | 0.5 (0.0) | 1.3 (0.0) | 2.8 (0.2) | 1.2 (0.3) | 0.7 (0.0) | 1.3 (0.0) |
| 48 | Skilled Birth Attendance | 3.8 (0.3) | 2.8 (0.8) | 0.6 (0.0) | 1.9 (0.0) | 2.9 (0.3) | 3.6 (1.0) | 0.7 (0.0) | 1.6 (0.0) |
| 49 | Unmet Need for Spacing | -3.3 (0.1) | 0.2 (0.1) | 0.2 (0.0) | 0.3 (0.0) | -2.8 (0.1) | 0.3 (0.1) | 0.1 (0.0) | 0.2 (0.0) |
|  | **Morbidity and Mortality** |  |  |  |  |  |  |  |  |
| 50 | Diarrhoea [Children] | -3.1 (0.1) | 0.2 (0.0) | 0.2 (0.0) | 0.6 (0.0) | -2.9 (0.1) | 0.2 (0.1) | 0.2 (0.0) | 0.5 (0.0) |
| 51 | Elevated Blood Pressure or On Medication [Men] | -1.6 (0.1) | 0.1 (0.0) | 0.1 (0.0) | 0.3 (0.0) | -1.7 (0.1) | 0.1 (0.0) | 0.1 (0.0) | 0.2 (0.0) |
| 52 | Elevated Blood Pressure or On Medication [Women] | -2.0 (0.1) | 0.1 (0.0) | 0.0 (0.0) | 0.2 (0.0) | -2.0 (0.0) | 0.1 (0.0) | 0.1 (0.0) | 0.2 (0.0) |
| 53 | High Blood Sugar [Men] | -3.1 (0.1) | 0.1 (0.0) | 0.1 (0.0) | 0.3 (0.0) | -3.2 (0.0) | 0.0 (0.0) | 0.1 (0.0) | 0.2 (0.0) |
| 54 | High Blood Sugar [Women] | -3.3 (0.1) | 0.1 (0.0) | 0.1 (0.0) | 0.2 (0.0) | -2.9 (0.0) | 0.1 (0.0) | 0.1 (0.0) | 0.2 (0.0) |
| 55 | High or Very High Blood Sugar or On Medication [Men] | -2.4 (0.1) | 0.1 (0.0) | 0.1 (0.0) | 0.2 (0.0) | -2.4 (0.0) | 0.1 (0.0) | 0.1 (0.0) | 0.3 (0.0) |
| 56 | High or Very High Blood Sugar or On Medication [Women] | -2.6 (0.1) | 0.1 (0.0) | 0.1 (0.0) | 0.2 (0.0) | -2.8 (0.0) | 0.1 (0.0) | 0.1 (0.0) | 0.2 (0.0) |
| 57 | Mildly Elevated Blood Pressure [Men] | -2.0 (0.1) | 0.1 (0.0) | 0.1 (0.0) | 0.3 (0.0) | -2.1 (0.1) | 0.1 (0.0) | 0.1 (0.0) | 0.2 (0.0) |
| 58 | Mildly Elevated Blood Pressure [Women] | -2.5 (0.1) | 0.1 (0.0) | 0.1 (0.0) | 0.2 (0.0) | -2.6 (0.1) | 0.1 (0.0) | 0.1 (0.0) | 0.2 (0.0) |
| 59 | Moderate or Severe Blood Pressure [Men] | -3.5 (0.1) | 0.2 (0.1) | 0.1 (0.0) | 0.4 (0.0) | -3.5 (0.1) | 0.2 (0.1) | 0.1 (0.0) | 0.2 (0.1) |
| 60 | Moderate or Severe Blood Pressure [Women] | -3.9 (0.1) | 0.2 (0.0) | 0.1 (0.0) | 0.3 (0.0) | -3.7 (0.1) | 0.1 (0.0) | 0.1 (0.0) | 0.2 (0.0) |
| 61 | Probability of Dying before Five Years | -3.8 (0.1) | 0.2 (0.1) | 0.1 (0.0) | 0.3 (0.0) | -3.5 (0.1) | 0.2 (0.1) | 0.1 (0.0) | 0.3 (0.0) |
| 62 | Probability of Dying before One Year | -3.9 (0.1) | 0.2 (0.1) | 0.1 (0.0) | 0.4 (0.0) | -3.6 (0.1) | 0.2 (0.1) | 0.0 (0.0) | 0.3 (0.0) |
| 63 | Probability of Dying within 28 Days | -4.3 (0.1) | 0.2 (0.1) | 0.1 (0.0) | 0.4 (0.0) | -4.1 (0.1) | 0.2 (0.1) | 0.0 (0.0) | 0.4 (0.0) |
| 64 | Risky Waist-to-hip Ratio [Women] | 0.6 (0.1) | 0.4 (0.1) | 0.2 (0.0) | 1.0 (0.0) |  |  |  |  |
| 65 | Very High Blood Sugar [Men] | -3.4 (0.1) | 0.2 (0.1) | 0.1 (0.0) | 0.3 (0.0) | -3.2 (0.1) | 0.1 (0.0) | 0.0 (0.0) | 0.3 (0.0) |
| 66 | Very High Blood Sugar [Women] | -3.6 (0.1) | 0.2 (0.1) | 0.1 (0.0) | 0.2 (0.0) | -3.7 (0.1) | 0.1 (0.0) | 0.1 (0.0) | 0.2 (0.0) |
|  | **Nutrition [Clinical/Anthropometry]** |  |  |  |  |  |  |  |  |
| 67 | Anaemia [Any - Adolescent Women] | 0.1 (0.1) | 0.4 (0.1) | 0.1 (0.0) | 0.3 (0.0) | 0.1 (0.1) | 0.3 (0.1) | 0.2 (0.0) | 0.3 (0.0) |
| 68 | Anaemia [Any - All Women] | 0.1 (0.1) | 0.4 (0.1) | 0.1 (0.0) | 0.3 (0.0) | 0.0 (0.1) | 0.3 (0.1) | 0.1 (0.0) | 0.2 (0.0) |
| 69 | Anaemia [Any - Pregnant Women] | -0.2 (0.1) | 0.3 (0.1) | 0.1 (0.0) | 0.4 (0.0) | -0.2 (0.1) | 0.2 (0.1) | 0.2 (0.0) | 0.3 (0.0) |
| 70 | Child Anaemia [Any] | 0.6 (0.1) | 0.4 (0.1) | 0.1 (0.0) | 0.5 (0.0) | 0.2 (0.1) | 0.4 (0.1) | 0.2 (0.0) | 0.4 (0.0) |
| 71 | Child Stunting | -0.8 (0.0) | 0.1 (0.0) | 0.1 (0.0) | 0.3 (0.0) | -0.8 (0.1) | 0.1 (0.0) | 0.1 (0.0) | 0.2 (0.0) |
| 72 | Child Underweight | -1.2 (0.1) | 0.2 (0.1) | 0.1 (0.0) | 0.4 (0.0) | -1.0 (0.1) | 0.3 (0.1) | 0.1 (0.0) | 0.2 (0.0) |
| 73 | Child Wasting | -1.8 (0.1) | 0.1 (0.0) | 0.1 (0.0) | 0.5 (0.0) | -1.6 (0.1) | 0.2 (0.0) | 0.1 (0.0) | 0.3 (0.0) |
| 74 | Mild Anaemia [Children] | -1.0 (0.0) | 0.0 (0.0) | 0.0 (0.0) | 0.1 (0.0) | -1.0 (0.0) | 0.1 (0.0) | 0.0 (0.0) | 0.1 (0.0) |
| 75 | Mild Anaemia [Women] | -1.2 (0.0) | 0.1 (0.0) | 0.0 (0.0) | 0.1 (0.0) | -0.5 (0.1) | 0.1 (0.0) | 0.1 (0.0) | 0.1 (0.0) |
| 76 | Moderate Anaemia [Children] | -1.0 (0.1) | 0.3 (0.1) | 0.1 (0.0) | 0.4 (0.0) | -1.2 (0.1) | 0.4 (0.1) | 0.2 (0.0) | 0.3 (0.0) |
| 77 | Moderate Anaemia [Women] | -1.2 (0.1) | 0.4 (0.1) | 0.1 (0.0) | 0.2 (0.0) | -1.2 (0.1) | 0.2 (0.1) | 0.1 (0.0) | 0.2 (0.0) |
| 78 | Overweight Children | -3.6 (0.1) | 0.2 (0.1) | 0.2 (0.0) | 1.0 (0.0) | -4.2 (0.1) | 0.3 (0.1) | 0.2 (0.0) | 1.0 (0.1) |
| 79 | Overweight or Obese [Women] | -1.2 (0.1) | 0.4 (0.1) | 0.2 (0.0) | 0.3 (0.0) | -1.5 (0.1) | 0.3 (0.1) | 0.2 (0.0) | 0.4 (0.0) |
| 80 | Severe Anaemia [Children] | -4.8 (0.1) | 0.6 (0.2) | 0.2 (0.0) | 0.9 (0.1) | -5.1 (0.1) | 0.6 (0.2) | 0.3 (0.0) | 0.7 (0.1) |
| 81 | Severe Anaemia [Women] | -4.1 (0.1) | 0.4 (0.1) | 0.2 (0.0) | 0.5 (0.0) | -4.1 (0.1) | 0.2 (0.1) | 0.2 (0.0) | 0.5 (0.0) |
| 82 | Severe Stunting [Children] | -2.1 (0.1) | 0.1 (0.0) | 0.1 (0.0) | 0.4 (0.0) | -2.1 (0.1) | 0.2 (0.0) | 0.1 (0.0) | 0.3 (0.0) |
| 83 | Severe Underweight [Children] | -2.7 (0.1) | 0.2 (0.1) | 0.1 (0.0) | 0.6 (0.0) | -2.7 (0.1) | 0.3 (0.1) | 0.1 (0.0) | 0.3 (0.0) |
| 84 | Severe Wasting [Children] | -3.0 (0.1) | 0.1 (0.0) | 0.2 (0.0) | 0.9 (0.0) | -2.9 (0.1) | 0.1 (0.0) | 0.2 (0.0) | 0.5 (0.0) |
| 85 | Underweight [Women] | -2.0 (0.1) | 0.4 (0.1) | 0.1 (0.0) | 0.3 (0.0) | -1.7 (0.1) | 0.3 (0.1) | 0.1 (0.0) | 0.2 (0.0) |
|  | **Nutrition [Diet]** |  |  |  |  |  |  |  |  |
| 86 | Adequate Diet [Breastfed Children] | -2.2 (0.1) | 0.3 (0.1) | 0.1 (0.0) | 0.9 (0.1) | -2.6 (0.1) | 0.6 (0.2) | 0.2 (0.0) | 0.9 (0.1) |
| 87 | Adequate Diet [Non-breastfed Children] | -2.3 (0.2) | 0.5 (0.2) | 0.1 (0.1) | 1.6 (0.4) | -2.4 (0.2) | 0.9 (0.3) | 0.2 (0.1) | 1.3 (0.3) |
| 88 | Adequate Diet [Total] | -2.2 (0.1) | 0.3 (0.1) | 0.1 (0.0) | 0.8 (0.1) | -2.5 (0.1) | 0.6 (0.2) | 0.2 (0.0) | 0.8 (0.1) |
| 89 | Early Breastfeeding Initiation | 0.0 (0.1) | 0.5 (0.2) | 0.2 (0.0) | 1.4 (0.0) | 0.0 (0.1) | 0.5 (0.1) | 0.2 (0.0) | 1.2 (0.0) |
| 90 | ICDS Supplementary Nutrition | 1.2 (0.2) | 1.6 (0.4) | 0.5 (0.0) | 1.3 (0.0) | 0.3 (0.2) | 1.6 (0.4) | 0.5 (0.0) | 1.4 (0.0) |
| 91 | Iodized Salt Intake | 5.6 (0.3) | 2.0 (0.5) | 1.1 (0.1) | 5.8 (0.1) | 5.4 (0.3) | 3.1 (0.8) | 1.3 (0.1) | 5.5 (0.1) |
| 92 | Exclusive Breastfeeding [Under 6 Months] | 0.7 (0.1) | 0.2 (0.1) | 0.1 (0.0) | 0.4 (0.1) | 0.3 (0.1) | 0.2 (0.1) | 0.2 (0.0) | 0.5 (0.1) |
| 93 | Receiving Solid/Semi-solid Food [6-8 Months] | 0.2 (0.1) | 0.4 (0.1) | 0.1 (0.0) | 0.5 (0.1) | 0.0 (0.1) | 0.5 (0.1) | 0.1 (0.0) | 0.3 (0.1) |
| 94 | Zero Food [Children] | -2.1 (0.1) | 0.3 (0.1) | 0.2 (0.0) | 0.7 (0.0) | -2.0 (0.1) | 0.2 (0.1) | 0.1 (0.0) | 0.5 (0.0) |
|  | **Social Infrastructure** |  |  |  |  |  |  |  |  |
| 95 | Access to Electricity | 7.3 (0.3) | 3.1 (0.9) | 1.4 (0.1) | 6.4 (0.1) | 6.0 (0.4) | 6.5 (1.8) | 1.9 (0.2) | 5.6 (0.1) |
| 96 | Clean Cooking Fuel | 1.1 (0.4) | 4.6 (1.3) | 1.6 (0.1) | 4.3 (0.0) | -0.4 (0.4) | 4.6 (1.4) | 2.0 (0.1) | 6.8 (0.1) |
| 97 | Death Registration | 1.7 (0.3) | 2.1 (0.6) | 0.2 (0.0) | 0.6 (0.0) |  |  |  |  |
| 98 | Handwashing Facilities | 2.1 (0.2) | 1.9 (0.5) | 0.8 (0.0) | 2.7 (0.0) | 1.2 (0.3) | 2.4 (0.6) | 0.8 (0.0) | 2.7 (0.0) |
| 99 | Hygienic Protection Methods [Menstruation] | 2.5 (0.2) | 1.8 (0.5) | 0.4 (0.0) | 1.3 (0.0) | 1.2 (0.2) | 1.7 (0.4) | 0.4 (0.0) | 1.4 (0.0) |
| 100 | Improved Sanitation Facility | 1.9 (0.2) | 1.9 (0.5) | 0.5 (0.0) | 1.8 (0.0) | 0.8 (0.3) | 3.2 (0.9) | 0.8 (0.1) | 2.9 (0.0) |
| 101 | Improved Source of Drinking Water | 6.5 (0.5) | 6.0 (1.8) | 4.3 (0.5) | 11.5 (0.2) | 4.5 (0.5) | 4.7 (1.3) | 3.4 (0.4) | 8.8 (0.1) |
| 102 | Internet Usage [Women] | -0.5 (0.1) | 0.7 (0.2) | 0.3 (0.0) | 0.8 (0.0) |  |  |  |  |
| 103 | Private Latrine | 2.3 (0.2) | 2.2 (0.6) | 0.6 (0.0) | 1.8 (0.0) | 1.2 (0.3) | 3.3 (0.9) | 1.0 (0.1) | 3.0 (0.0) |
| 104 | Safe Stool Disposal | -0.2 (0.2) | 1.2 (0.3) | 0.4 (0.0) | 1.7 (0.0) | 0.1 (0.3) | 2.8 (0.8) | 0.6 (0.0) | 1.8 (0.0) |
| 105 | Women with Personal Mobile Phone | 0.7 (0.1) | 0.7 (0.2) | 0.2 (0.0) | 0.5 (0.0) | 0.2 (0.1) | 0.6 (0.2) | 0.2 (0.0) | 0.7 (0.0) |
| 106 | Alcohol Consumption [Men] | -1.5 (0.2) | 0.9 (0.3) | 0.3 (0.0) | 0.4 (0.0) | -0.7 (0.1) | 0.7 (0.2) | 0.3 (0.0) | 0.7 (0.0) |
| 107 | Alcohol Consumption [Women] | -5.5 (0.3) | 2.3 (0.6) | 0.7 (0.1) | 1.4 (0.0) | -6.0 (0.4) | 4.8 (1.3) | 1.8 (0.1) | 2.1 (0.1) |
| 108 | Child Marriage [Boy] | -2.0 (0.1) | 0.5 (0.2) | 0.2 (0.0) | 0.6 (0.1) | -1.9 (0.1) | 0.5 (0.2) | 0.2 (0.0) | 0.4 (0.1) |
| 109 | Child Marriage [Girl] | -1.8 (0.1) | 0.7 (0.2) | 0.3 (0.0) | 0.4 (0.0) | -1.6 (0.1) | 0.5 (0.2) | 0.3 (0.0) | 0.4 (0.0) |
| 110 | Currently Working Women | -1.2 (0.1) | 0.3 (0.1) | 0.2 (0.0) | 0.6 (0.0) | -1.4 (0.1) | 0.3 (0.1) | 0.2 (0.0) | 0.7 (0.0) |
| 111 | Female School Attendance | 1.3 (0.1) | 0.4 (0.1) | 0.1 (0.0) | 0.2 (0.0) | 1.2 (0.1) | 0.4 (0.1) | 0.1 (0.0) | 0.3 (0.0) |
| 112 | High School Matriculation [Men] | 0.1 (0.1) | 0.4 (0.1) | 0.1 (0.0) | 0.6 (0.0) | -0.1 (0.1) | 0.3 (0.1) | 0.1 (0.0) | 0.7 (0.0) |
| 113 | High School Matriculation [Women] | -0.3 (0.1) | 0.4 (0.1) | 0.2 (0.0) | 0.5 (0.0) | -0.6 (0.1) | 0.5 (0.1) | 0.2 (0.0) | 0.8 (0.0) |
| 114 | Intimate Partner Violence [Against Women] | -1.7 (0.1) | 0.7 (0.2) | 0.2 (0.0) | 0.9 (0.0) | -1.3 (0.1) | 0.8 (0.2) | 0.1 (0.0) | 0.7 (0.0) |
| 115 | Literacy [Men] | 2.4 (0.1) | 0.4 (0.1) | 0.2 (0.0) | 0.8 (0.0) | 2.2 (0.1) | 0.6 (0.2) | 0.2 (0.0) | 0.7 (0.0) |
| 116 | Literacy [Women] | 1.5 (0.1) | 0.7 (0.2) | 0.2 (0.0) | 0.5 (0.0) | 1.4 (0.2) | 0.8 (0.2) | 0.3 (0.0) | 0.6 (0.0) |
| 117 | Population below 15 Years | -1.1 (0.0) | 0.1 (0.0) | 0.0 (0.0) | 0.0 (0.0) | -1.0 (0.0) | 0.0 (0.0) | 0.0 (0.0) | 0.0 (0.0) |
| 118 | Sexual Violence [Young Women] | -5.5 (0.2) | 0.5 (0.2) | 0.3 (0.2) | 1.6 (0.4) | -4.9 (0.2) | 0.7 (0.3) | 0.1 (0.1) | 0.8 (0.2) |
| 119 | Teenage Pregnancy | -3.2 (0.1) | 0.6 (0.2) | 0.2 (0.0) | 0.3 (0.0) | -2.9 (0.1) | 0.4 (0.1) | 0.2 (0.0) | 0.3 (0.0) |
| 120 | Tobacco Consumption [Women] | -4.1 (0.3) | 4.3 (1.1) | 0.8 (0.0) | 1.0 (0.0) | -3.2 (0.3) | 3.4 (0.9) | 0.5 (0.0) | 0.7 (0.0) |
| 121 | Tobacco Use [Men] | -0.8 (0.2) | 0.8 (0.2) | 0.1 (0.0) | 0.2 (0.0) | -0.2 (0.1) | 0.7 (0.2) | 0.1 (0.0) | 0.4 (0.0) |
| 122 | Women Participation in Household Decisions | 2.9 (0.1) | 0.5 (0.2) | 0.2 (0.0) | 1.3 (0.1) | 2.4 (0.1) | 0.5 (0.2) | 0.2 (0.0) | 0.9 (0.0) |

*Note*. The following indicators were excluded from 2016 estimates due to data limitations: Measles-Containing Vaccine (2nd Dose), Rotavirus Vaccine (3 Doses), Risky Waist-to-Hip Ratio (Women), Death Registration, Internet Usage (Women). Estimates and standard errors are based on the posterior means and standard deviations from MCMC chains.

**Supplementary Table 6. Sample Sizes for 122 Indicators, 2021 and 2016**

| **Indicator No** | **Category-Indicator Label** | **2021** | | | | **2016** | | | |
| --- | --- | --- | --- | --- | --- | --- | --- | --- | --- |
|  |  | **Individual n** | **Cluster n** | **District N** | **State N** | **Individual n** | **Cluster n** | **District N** | **State N** |
|  | **Socio-Economic Profile** |  |  |  |  |  |  |  |  |
| 1 | Population with BPL cards | 2839275 | 30170 | 720 | 36 | 2858430 | 28470 | 720 | 36 |
|  | **Health Care** |  |  |  |  |  |  |  |  |
| 2 | Acute Respiratory Infection [All Children] | 223862 | 29757 | 720 | 36 | 247166 | 28268 | 720 | 36 |
| 3 | Acute Respiratory Infection [Children Getting Treatment - Facility] | 27676 | 13922 | 719 | 36 | 31992 | 14971 | 715 | 36 |
| 4 | Diarrhoea [Received ORS] | 15302 | 9499 | 718 | 36 | 22413 | 12126 | 716 | 36 |
| 5 | Diarrhoea [Received Zinc] | 14914 | 9316 | 718 | 36 | 21786 | 11929 | 716 | 36 |
| 6 | Diarrhoea Treatment [Facility] | 15334 | 9514 | 718 | 36 | 22463 | 12145 | 716 | 36 |
| 7 | DPT Vaccination [3 Doses] | 43377 | 21762 | 720 | 36 | 48937 | 22156 | 720 | 36 |
| 8 | Full Vaccination | 43126 | 21715 | 720 | 36 | 48577 | 22089 | 720 | 36 |
| 9 | Full Vaccination [Vaccination Card] | 37737 | 20379 | 720 | 36 | 30796 | 17662 | 720 | 36 |
| 10 | Health Insurance [Any] | 2829625 | 30170 | 720 | 36 | 2846790 | 28470 | 720 | 36 |
| 11 | Hepatitis B Vaccine [3 Doses] | 38509 | 20469 | 720 | 36 | 48479 | 22083 | 720 | 36 |
| 12 | ICDS Benefits [Children] | 271924 | 29923 | 720 | 36 | 295048 | 28344 | 720 | 36 |
| 13 | Low Birth Weight | 203095 | 29648 | 720 | 36 | 186918 | 27853 | 720 | 36 |
| 14 | Measles-Containing Vaccine [First Dose] | 43197 | 21731 | 720 | 36 | 48808 | 22127 | 720 | 36 |
| 15 | Measles-Containing Vaccine [Second Dose] | 43837 | 21995 | 720 | 36 |  |  |  |  |
| 16 | Polio Vaccination [3 Doses] | 43451 | 21778 | 720 | 36 | 49096 | 22184 | 720 | 36 |
| 17 | Rotavirus Vaccine [3 Doses] | 43006 | 21688 | 720 | 36 |  |  |  |  |
| 18 | Vitamin A Dose | 97287 | 27755 | 720 | 36 | 108856 | 26938 | 720 | 36 |
| 19 | Zero Dose [Child Immunization] | 43377 | 21762 | 720 | 36 | 48937 | 22156 | 720 | 36 |
|  | **Maternal Health and Family Planning** |  |  |  |  |  |  |  |  |
| 20 | Antenatal Care Visit [Four or More] | 170107 | 29708 | 720 | 36 | 182937 | 28251 | 720 | 36 |
| 21 | Antenatal Care Visit [First Trimester] | 172426 | 29751 | 720 | 36 | 184749 | 28268 | 720 | 36 |
| 22 | Birth Registration | 227995 | 29784 | 720 | 36 | 255239 | 28320 | 720 | 36 |
| 23 | Birth Weight Recorded | 224218 | 29757 | 720 | 36 | 247253 | 28268 | 720 | 36 |
| 24 | Caesarean Section Delivery | 224218 | 29757 | 720 | 36 | 247253 | 28268 | 720 | 36 |
| 25 | Caesarean Section in Private Sector | 48032 | 17997 | 717 | 36 | 50942 | 17869 | 715 | 36 |
| 26 | Caesarean Section in Public Sector | 145047 | 27740 | 717 | 36 | 135077 | 26056 | 720 | 36 |
| 27 | Childbirths in Public Facility | 224218 | 29757 | 720 | 36 | 247253 | 28268 | 720 | 36 |
| 28 | Condom | 512408 | 30159 | 720 | 36 | 498707 | 28464 | 720 | 36 |
| 29 | Family Planning [Any Methods by Women] | 512408 | 30159 | 720 | 36 | 498707 | 28464 | 720 | 36 |
| 30 | Family Planning [Modern] | 512408 | 30159 | 720 | 36 | 498707 | 28464 | 720 | 36 |
| 31 | Family Planning [Unmet Need] | 512408 | 30159 | 720 | 36 | 498707 | 28464 | 720 | 36 |
| 32 | Family Planning Services Quality [Family Planning Counselling] | 373711 | 30150 | 720 | 36 | 437617 | 28461 | 720 | 36 |
| 33 | Female Sterilization | 512408 | 30159 | 720 | 36 | 498707 | 28464 | 720 | 36 |
| 34 | Family Planning Services Quality [Side Effects Counselling] | 69075 | 24878 | 720 | 36 | 62038 | 22833 | 720 | 36 |
| 35 | Home Delivery by Skilled Health Personnel | 224218 | 29757 | 720 | 36 | 247253 | 28268 | 720 | 36 |
| 36 | Injectables | 512408 | 30159 | 720 | 36 | 498707 | 28464 | 720 | 36 |
| 37 | Institutional Childbirth | 232920 | 29770 | 720 | 36 | 258950 | 28276 | 720 | 36 |
| 38 | Iron Folic Acid [100 days or More] | 172426 | 29751 | 720 | 36 | 184749 | 28268 | 720 | 36 |
| 39 | Iron Folic Acid [180 days or More] | 172426 | 29751 | 720 | 36 | 184749 | 28268 | 720 | 36 |
| 40 | IUD/PPIUD | 512408 | 30159 | 720 | 36 | 498707 | 28464 | 720 | 36 |
| 41 | Male Sterilization | 512408 | 30159 | 720 | 36 | 498707 | 28464 | 720 | 36 |
| 42 | Maternal Care Quality [Postpartum] | 77939 | 24157 | 720 | 36 | 90699 | 24211 | 719 | 36 |
| 43 | Mother and Child Protection Card | 165941 | 29640 | 720 | 36 | 160474 | 27892 | 720 | 36 |
| 44 | Neonatal Tetanus | 169491 | 29716 | 720 | 36 | 181733 | 28248 | 720 | 36 |
| 45 | Pill | 512408 | 30159 | 720 | 36 | 498707 | 28464 | 720 | 36 |
| 46 | Postnatal Care [Mothers] | 172426 | 29751 | 720 | 36 | 184749 | 28268 | 720 | 36 |
| 47 | Pregnancy Registration | 172426 | 29751 | 720 | 36 | 184749 | 28268 | 720 | 36 |
| 48 | Skilled Birth Attendance | 224218 | 29757 | 720 | 36 | 247253 | 28268 | 720 | 36 |
| 49 | Unmet Need for Spacing | 512408 | 30159 | 720 | 36 | 498707 | 28464 | 720 | 36 |
|  | **Morbidity and Mortality** |  |  |  |  |  |  |  |  |
| 50 | Diarrhoea [Children] | 223785 | 29757 | 720 | 36 | 246695 | 28268 | 720 | 36 |
| 51 | Elevated Blood Pressure or On Medication [Men] | 542272 | 29730 | 720 | 36 | 96220 | 9774 | 718 | 36 |
| 52 | Elevated Blood Pressure or On Medication [Women] | 653788 | 29829 | 720 | 36 | 660561 | 28351 | 720 | 36 |
| 53 | High Blood Sugar [Men] | 586356 | 30089 | 720 | 36 | 108209 | 9863 | 718 | 36 |
| 54 | High Blood Sugar [Women] | 693023 | 30113 | 720 | 36 | 683582 | 28456 | 720 | 36 |
| 55 | High or Very High Blood Sugar or On Medication [Men] | 585263 | 30088 | 720 | 36 | 108209 | 9863 | 718 | 36 |
| 56 | High or Very High Blood Sugar or On Medication [Women] | 690703 | 30113 | 720 | 36 | 683582 | 28456 | 720 | 36 |
| 57 | Mildly Elevated Blood Pressure [Men] | 542303 | 29730 | 720 | 36 | 104226 | 9783 | 718 | 36 |
| 58 | Mildly Elevated Blood Pressure [Women] | 653883 | 29828 | 720 | 36 | 660580 | 28351 | 720 | 36 |
| 59 | Moderate or Severe Blood Pressure [Men] | 542303 | 29867 | 720 | 36 | 96222 | 9774 | 718 | 36 |
| 60 | Moderate or Severe Blood Pressure [Women] | 653882 | 29829 | 720 | 36 | 660580 | 28351 | 720 | 36 |
| 61 | Probability of Dying before Five Years | 232920 | 29770 | 720 | 36 | 259108 | 28279 | 720 | 36 |
| 62 | Probability of Dying before One Year | 232920 | 29770 | 720 | 36 | 259108 | 28279 | 720 | 36 |
| 63 | Probability of Dying within 28 Days | 232920 | 29770 | 720 | 36 | 259108 | 28279 | 720 | 36 |
| 64 | Risky Waist-to-hip Ratio [Women] | 685935 | 30161 | 720 | 36 |  |  |  |  |
| 65 | Very High Blood Sugar [Men] | 586356 | 30089 | 720 | 36 | 108209 | 9863 | 718 | 36 |
| 66 | Very High Blood Sugar [Women] | 690748 | 30113 | 720 | 36 | 683582 | 28456 | 720 | 36 |
|  | **Nutrition [Clinical/Anthropometry]** |  |  |  |  |  |  |  |  |
| 67 | Anaemia [Any - Adolescent Women] | 122480 | 28655 | 720 | 36 | 121516 | 27354 | 720 | 36 |
| 68 | Anaemia [Any - All Women] | 724115 | 30161 | 720 | 36 | 683648 | 28457 | 720 | 36 |
| 69 | Anaemia [Any - Pregnant Women] | 28408 | 17285 | 720 | 36 | 31790 | 17864 | 718 | 36 |
| 70 | Child Anaemia [Any] | 183880 | 29235 | 720 | 36 | 209074 | 28036 | 720 | 36 |
| 71 | Child Stunting | 206025 | 29505 | 720 | 36 | 224560 | 28111 | 720 | 36 |
| 72 | Child Underweight | 210524 | 29551 | 720 | 36 | 224560 | 28111 | 720 | 36 |
| 73 | Child Wasting | 201687 | 29440 | 720 | 36 | 224560 | 28111 | 720 | 36 |
| 74 | Mild Anaemia [Children] | 183880 | 29235 | 720 | 36 | 209074 | 28036 | 720 | 36 |
| 75 | Mild Anaemia [Women] | 724115 | 30161 | 720 | 36 | 683648 | 28457 | 720 | 36 |
| 76 | Moderate Anaemia [Children] | 183880 | 29235 | 720 | 36 | 209074 | 28036 | 720 | 36 |
| 77 | Moderate Anaemia [Women] | 705894 | 30157 | 720 | 36 | 667692 | 28457 | 720 | 36 |
| 78 | Overweight Children | 201610 | 29438 | 720 | 36 | 224498 | 28111 | 720 | 36 |
| 79 | Overweight or Obese [Women] | 671744 | 30117 | 720 | 36 | 653947 | 28458 | 720 | 36 |
| 80 | Severe Anaemia [Children] | 183880 | 29235 | 720 | 36 | 209074 | 28083 | 720 | 36 |
| 81 | Severe Anaemia [Women] | 232920 | 29770 | 720 | 36 | 683648 | 28457 | 720 | 36 |
| 82 | Severe Stunting [Children] | 206015 | 29505 | 720 | 36 | 224558 | 28111 | 720 | 36 |
| 83 | Severe Underweight [Children] | 210524 | 29551 | 720 | 36 | 224560 | 28111 | 720 | 36 |
| 84 | Severe Wasting [Children] | 201687 | 29440 | 720 | 36 | 224560 | 28111 | 720 | 36 |
| 85 | Underweight [Women] | 671744 | 30117 | 720 | 36 | 653947 | 28458 | 720 | 36 |
|  | **Nutrition [Diet]** |  |  |  |  |  |  |  |  |
| 86 | Adequate Diet [Breastfed Children] | 53068 | 23427 | 720 | 36 | 59950 | 23551 | 720 | 36 |
| 87 | Adequate Diet [Non-breastfed Children] | 8032 | 6552 | 711 | 36 | 9692 | 7577 | 700 | 36 |
| 88 | Adequate Diet [Total] | 60953 | 24802 | 720 | 36 | 69496 | 24765 | 720 | 36 |
| 89 | Early Breastfeeding Initiation | 117974 | 28749 | 720 | 36 | 130667 | 27667 | 720 | 36 |
| 90 | ICDS Supplementary Nutrition | 172426 | 29751 | 720 | 36 | 184749 | 28268 | 720 | 36 |
| 91 | Iodized Salt Intake | 2843917 | 30170 | 720 | 36 | 2863760 | 28470 | 720 | 36 |
| 92 | Exclusive Breastfeeding [Under 6 Months] | 21141 | 14130 | 720 | 36 | 22587 | 14643 | 719 | 36 |
| 93 | Receiving Solid/Semi-solid Food [6-8 Months] | 10777 | 8777 | 720 | 36 | 13052 | 10068 | 715 | 36 |
| 94 | Zero Food [Children] | 62009 | 24999 | 720 | 36 | 71146 | 24977 | 720 | 36 |
|  | **Social Infrastructure** |  |  |  |  |  |  |  |  |
| 95 | Access to Electricity | 2843917 | 30170 | 720 | 36 | 2863760 | 28470 | 720 | 36 |
| 96 | Clean Cooking Fuel | 2843917 | 30170 | 720 | 36 | 2863760 | 28470 | 720 | 36 |
| 97 | Death Registration | 81340 | 27018 | 720 | 36 |  |  |  |  |
| 98 | Handwashing Facilities | 2748448 | 30169 | 720 | 36 | 2771171 | 28452 | 720 | 36 |
| 99 | Hygienic Protection Methods [Menstruation] | 239575 | 30007 | 720 | 36 | 245031 | 28373 | 720 | 36 |
| 100 | Improved Sanitation Facility | 2843910 | 30170 | 720 | 36 | 2863760 | 28470 | 720 | 36 |
| 101 | Improved Source of Drinking Water | 2843917 | 30170 | 720 | 36 | 2863760 | 28470 | 720 | 36 |
| 102 | Internet Usage [Women] | 108785 | 9107 | 720 | 36 |  |  |  |  |
| 103 | Private Latrine | 2843917 | 30170 | 720 | 36 | 2863760 | 28470 | 720 | 36 |
| 104 | Safe Stool Disposal | 85204 | 26998 | 720 | 36 | 185931 | 28267 | 720 | 36 |
| 105 | Women with Personal Mobile Phone | 108785 | 9107 | 720 | 36 | 122188 | 9874 | 718 | 36 |
| 106 | Alcohol Consumption [Men] | 700561 | 30168 | 720 | 36 | 103398 | 9870 | 718 | 36 |
| 107 | Alcohol Consumption [Women] | 747176 | 30166 | 720 | 36 | 698379 | 28467 | 720 | 36 |
| 108 | Child Marriage [Boy] | 14360 | 6951 | 719 | 36 | 16136 | 7821 | 717 | 36 |
| 109 | Child Marriage [Girl] | 118700 | 29023 | 720 | 36 | 122745 | 27752 | 720 | 36 |
| 110 | Currently Working Women | 98184 | 9101 | 720 | 36 | 109956 | 9868 | 718 | 36 |
| 111 | Female School Attendance | 1293773 | 30170 | 720 | 36 | 1269781 | 28469 | 720 | 36 |
| 112 | High School Matriculation [Men] | 93267 | 9100 | 720 | 36 | 111978 | 9873 | 718 | 36 |
| 113 | High School Matriculation [Women] | 724115 | 30161 | 720 | 36 | 698379 | 28467 | 720 | 36 |
| 114 | Intimate Partner Violence [Against Women] | 63815 | 9101 | 720 | 36 | 65635 | 9861 | 718 | 36 |
| 115 | Literacy [Men] | 93267 | 9100 | 720 | 36 | 111978 | 9873 | 718 | 36 |
| 116 | Literacy [Women] | 724115 | 30161 | 720 | 36 | 698379 | 28467 | 720 | 36 |
| 117 | Population below 15 Years | 2843917 | 30170 | 720 | 36 | 2863760 | 28470 | 720 | 36 |
| 118 | Sexual Violence [Young Women] | 20778 | 8038 | 720 | 36 | 24140 | 8926 | 716 | 36 |
| 119 | Teenage Pregnancy | 122480 | 28655 | 720 | 36 | 124657 | 27496 | 720 | 36 |
| 120 | Tobacco Consumption [Women] | 724115 | 30161 | 720 | 36 | 698379 | 28467 | 720 | 36 |
| 121 | Tobacco Use [Men] | 700561 | 30168 | 720 | 36 | 103398 | 9870 | 718 | 36 |
| 122 | Women Participation in Household Decisions | 76910 | 9104 | 720 | 36 | 86694 | 9871 | 718 | 36 |

*Note*. The following indicators were excluded from 2016 estimates due to data limitations: Measles-Containing Vaccine (2nd Dose), Rotavirus Vaccine (3 Doses), Risky Waist-to-Hip Ratio (Women), Death Registration, Internet Usage (Women).

**Supplementary Table 7. Headcount Estimates for 122 Indicators, 2021 and 2016**

| **Indicator No** | **Category-Indicator Label** | **Headcount (N)** | |
| --- | --- | --- | --- |
|  |  | **District Min - Max** | **PC Min - Max** |
|  | **Socio-Economic Profile** |  |  |
| 1 | Population with BPL cards | 3664-5100000 | 25385-3900000 |
|  | **Health Care** |  |  |
| 2 | Acute Respiratory Infection [All Children] | 7-33636 | 69-29434 |
| 3 | Acute Respiratory Infection [Children Getting Treatment - Facility] | 349-951869 | 4407-453430 |
| 4 | Diarrhoea [Received ORS] | 372-686400 | 3111-316679 |
| 5 | Diarrhoea [Received Zinc] | 181-305038 | 896-168907 |
| 6 | Diarrhoea Treatment [Facility] | 321-832234 | 3813-432221 |
| 7 | DPT Vaccination [3 Doses] | 77-170309 | 665-90791 |
| 8 | Full Vaccination | 73-163070 | 626-77211 |
| 9 | Full Vaccination [Vaccination Card] | 76-162995 | 671-89323 |
| 10 | Health Insurance [Any] | 947-5700000 | 8380-4500000 |
| 11 | Hepatitis B Vaccine [3 Doses] | 75-167665 | 651-84802 |
| 12 | ICDS Benefits [Children] | 181-719860 | 2677-327731 |
| 13 | Low Birth Weight | 67-195700 | 546-114631 |
| 14 | Measles-Containing Vaccine [First Dose] | 79-172791 | 670-91617 |
| 15 | Supplementary Table | 76-116874 | 135-64272 |
| 16 | Polio Vaccination [3 Doses] | 74-167821 | 632-79928 |
| 17 | Rotavirus Vaccine [3 Doses] | 1-90577 | 16-58829 |
| 18 | Vitamin A Dose | 134-292576 | 805-156296 |
| 19 | Zero Dose [Child Immunization] | 5-12514 | 46-9362 |
|  | **Maternal Health and Family Planning** |  |  |
| 20 | Antenatal Care Visit [Four or More] | 192-752596 | 4429-324287 |
| 21 | Antenatal Care Visit [First Trimester] | 297-748357 | 4539-336283 |
| 22 | Birth Registration | 536-987297 | 4926-454682 |
| 23 | Birth Weight Recorded | 523-1100000 | 4956-485892 |
| 24 | Caesarean Section Delivery | 46-363283 | 1473-161387 |
| 25 | Caesarean Section in Private Sector | 4-230851 | 32-105036 |
| 26 | Caesarean Section in Public Sector | 31-182037 | 783-70401 |
| 27 | Childbirths in Public Facility | 477-658730 | 3498-391402 |
| 28 | Condom | 68-282200 | 593-230103 |
| 29 | Family Planning [Any Methods by Women] | 1107-2000000 | 8415-848249 |
| 30 | Family Planning [Modern] | 798-1600000 | 4811-632149 |
| 31 | Family Planning [Unmet Need] | 80-295905 | 1851-141100 |
| 32 | Family Planning Services Quality [Family Planning Counselling] | 495-1200000 | 3427-447411 |
| 33 | Female Sterilization | 369-1200000 | 3130-456846 |
| 34 | Family Planning Services Quality [Side Effects Counselling] | 1430-1800000 | 13565-853342 |
| 35 | Home Delivery by Skilled Health Personnel | 0-27746 | 0-23228 |
| 36 | Injectables | 6-29459 | 11-22733 |
| 37 | Institutional Childbirth | 494-1000000 | 4965-494726 |
| 38 | Iron Folic Acid [100 days or More] | 91-517163 | 1664-211993 |
| 39 | Iron Folic Acid [180 days or More] | 22-323017 | 741-162206 |
| 40 | IUD/PPIUD | 53-64581 | 124-56579 |
| 41 | Male Sterilization | 0-30097 | 10-48015 |
| 42 | Maternal Care Quality [Postpartum] | 503-909200 | 4574-411182 |
| 43 | Mother and Child Protection Card | 500-885385 | 4095-403164 |
| 44 | Neonatal Tetanus | 446-840224 | 4477-381825 |
| 45 | Pill | 19-725750 | 171-270053 |
| 46 | Postnatal Care [Mothers] | 337-835606 | 4357-378539 |
| 47 | Pregnancy Registration | 494-906654 | 4525-408633 |
| 48 | Skilled Birth Attendance | 493-1000000 | 4965-477470 |
| 49 | Unmet Need for Spacing | 60-110742 | 1165-80869 |
|  | **Morbidity and Mortality** |  |  |
| 50 | Diarrhoea [Children] | 23-159419 | 123-153077 |
| 51 | Elevated Blood Pressure or On Medication [Men] | 732-925727 | 2388-336344 |
| 52 | Elevated Blood Pressure or On Medication [Women] | 446-474607 | 2061-182773 |
| 53 | High Blood Sugar [Men] | 101-308991 | 1056-125657 |
| 54 | High Blood Sugar [Women] | 74-205042 | 830-85747 |
| 55 | High or Very High Blood Sugar or On Medication [Men] | 214-504182 | 1811-199900 |
| 56 | High or Very High Blood Sugar or On Medication [Women] | 156-378579 | 1866-160551 |
| 57 | Mildly Elevated Blood Pressure [Men] | 563-639596 | 1821-246323 |
| 58 | Mildly Elevated Blood Pressure [Women] | 320-325462 | 1460-135420 |
| 59 | Moderate or Severe Blood Pressure [Men] | 172-183020 | 366-70345 |
| 60 | Moderate or Severe Blood Pressure [Women] | 92-87461 | 381-34744 |
| 61 | Probability of Dying before Five Years | 11-45281 | 56-33495 |
| 62 | Probability of Dying before One Year | 9-38347 | 51-30074 |
| 63 | Probability of Dying within 28 Days | 4-27475 | 39-21817 |
| 64 | Risky Waist-to-hip Ratio [Women] | 1979-2700000 | 15638-897918 |
| 65 | Very High Blood Sugar [Men] | 76-153337 | 470-79157 |
| 66 | Very High Blood Sugar [Women] | 63-148540 | 622-71458 |
|  | **Nutrition [Clinical/Anthropometry]** |  |  |
| 67 | Anaemia [Any - Adolescent Women] | 269-380400 | 1063-165421 |
| 68 | Anaemia [Any - All Women] | 1391-2100000 | 5253-885090 |
| 69 | Anaemia [Any - Pregnant Women] | 44-73971 | 236-59615 |
| 70 | Child Anaemia [Any] | 325-616320 | 1935-418556 |
| 71 | Child Stunting | 111-403829 | 1485-286389 |
| 72 | Child Underweight | 86-351211 | 1307-274672 |
| 73 | Child Wasting | 87-273077 | 862-134789 |
| 74 | Mild Anaemia [Children] | 147-278901 | 1317-175847 |
| 75 | Mild Anaemia [Women] | 471-962559 | 3155-387326 |
| 76 | Moderate Anaemia [Children] | 107-308372 | 487-215789 |
| 77 | Moderate Anaemia [Women] | 411-1100000 | 2262-447932 |
| 78 | Overweight Children | 25-32806 | 255-17480 |
| 79 | Overweight or Obese [Women] | 496-1100000 | 6703-433727 |
| 80 | Severe Anaemia [Children] | 3-18877 | 31-14989 |
| 81 | Severe Anaemia [Women] | 40-104090 | 162-40194 |
| 82 | Severe Stunting [Children] | 42-183149 | 380-122767 |
| 83 | Severe Underweight [Children] | 35-104406 | 490-90847 |
| 84 | Severe Wasting [Children] | 38-82959 | 407-66069 |
| 85 | Underweight [Women] | 95-661973 | 1712-309520 |
|  | **Nutrition [Diet]** |  |  |
| 86 | Adequate Diet [Breastfed Children] | 19-46517 | 172-21528 |
| 87 | Adequate Diet [Non-breastfed Children] | 1852909 | 1349024 |
| 88 | Adequate Diet [Total] | 20-50296 | 182-24078 |
| 89 | Early Breastfeeding Initiation | 209-286406 | 1422-145307 |
| 90 | ICDS Supplementary Nutrition | 126-784988 | 4204-343327 |
| 91 | Iodized Salt Intake | 8169-11000000 | 68044-5300000 |
| 92 | Exclusive Breastfeeding [Under 6 Months] | 40-77359 | 462-48372 |
| 93 | Receiving Solid/Semi-solid Food [6-8 Months] | 14-36261 | 56-17449 |
| 94 | Zero Food [Children] | 16-72607 | 186-43814 |
|  | **Social Infrastructure** |  |  |
| 95 | Access to Electricity | 6976-14000000 | 74189-5700000 |
| 96 | Clean Cooking Fuel | 4076-13000000 | 43817-5600000 |
| 97 | Death Registration | 3133-13000000 | 72608-5500000 |
| 98 | Handwashing Facilities | 7187-12000000 | 67903-5300000 |
| 99 | Hygienic Protection Methods [Menstruation] | 672-1000000 | 5922-440309 |
| 100 | Improved Sanitation Facility | 8300-11000000 | 50962-5200000 |
| 101 | Improved Source of Drinking Water | 8746-13000000 | 67539-5700000 |
| 102 | Internet Usage [Women] | 983-2100000 | 11861-866199 |
| 103 | Private Latrine | 8423-11000000 | 54404-5300000 |
| 104 | Safe Stool Disposal | 316-585542 | 4201-266248 |
| 105 | Women with Personal Mobile Phone | 2109-2700000 | 18236-1000000 |
| 106 | Alcohol Consumption [Men] | 139-584692 | 140-481471 |
| 107 | Alcohol Consumption [Women] | 73-148449 | 204-221971 |
| 108 | Child Marriage [Boy] | 65-99913 | 181-78765 |
| 109 | Child Marriage [Girl] | 53-258925 | 79-110569 |
| 110 | Currently Working Women | 492-1200000 | 2541-533637 |
| 111 | Female School Attendance | 1417-2700000 | 18427-1100000 |
| 112 | High School Matriculation [Men] | 1432-3200000 | 15475-1100000 |
| 113 | High School Matriculation [Women] | 1006-2300000 | 15073-830118 |
| 114 | Intimate Partner Violence [Against Women] | 355-941294 | 642-365176 |
| 115 | Literacy [Men] | 2248-4400000 | 18968-1800000 |
| 116 | Literacy [Women] | 1852-3300000 | 20724-1300000 |
| 117 | Population below 15 Years | 1917-3000000 | 16355-1600000 |
| 118 | Sexual Violence [Young Women] | 7-25184 | 53-17833 |
| 119 | Teenage Pregnancy | 10-95587 | 51-41880 |
| 120 | Tobacco Consumption [Women] | 9-202689 | 223-231101 |
| 121 | Tobacco Use [Men] | 610-1500000 | 4631-605091 |
| 122 | Women Participation in Household Decisions | 1488-2600000 | 15030-1000000 |

**Supplementary Table 8. Estimated and Reported Prevalence for Validation**

| **IPI Indicator Name** | **IPI - All India Prevalence (%)** | **NFHS-5 Report/Factsheet Estimate** | **Notes** |
| --- | --- | --- | --- |
| Access to Electricity | 96.76 | 96.80 | Verified - No Difference |
| Rotavirus Vaccine [3 Doses] | 36.90 | 36.40 | Verified - Negligible Difference |
| Acute Respiratory Infection [All Children] | 2.80 | 2.80 | Verified - No Difference |
| Acute Respiratory Infection [Children Getting Treatment - Facility] | 69.20 | 69.00 | Verified - Negligible Difference |
| Adequate Diet [Breastfed Children] | 10.60 | 9.60 | Verified - Negligible Difference |
| Adequate Diet [Non-breastfed Children] | 12.50 | 14.30 | Verified - Negligible Difference |
| Adequate Diet [Total] | 10.90 | 12.30 | Verified - Negligible Difference |
| Alcohol Consumption [Men] | 17.50 | 18.80 | Verified - Negligible Difference |
| Alcohol Consumption [Women] | 0.70 | 0.70 | Verified - Negligible Difference |
| Anaemia [Any - Adolescent Women] | 59.10 | 59.10 | Verified - No Difference |
| Anaemia [Any - All Women] | 53.76 | - | Verification Not Possible - Multiple Estimation Variables |
| Anaemia [Any - Pregnant Women] | 49.67 | - | Verification Not Possible - Multiple Estimation Variables |
| Antenatal Care Visit [Four or More] | 59.50 | 58.10 | Verified - Negligible Difference |
| Antenatal Care Visit [First Trimester] | 70.19 | 70.00 | Verified - No Difference |
| Child Anaemia [Any] | 68.00 | 67.10 | Verified - Negligible Difference |
| Birth Registration | 89.06 | 89.10 | Verified - Negligible Difference |
| Birth Weight Recorded | 91.37 | 91.40 | Verified - No Difference |
| Caesarean Section Delivery | 21.70 | 21.50 | Verified - Negligible Difference |
| Child Marriage [Boy] | 18.15 | 20.30 | Verified - Negligible Difference |
| Caesarean Section in Private Sector | 47.80 | 47.80 | Verified - No Difference |
| Caesarean Section in Public Sector | 14.40 | 14.30 | Verified - No Difference |
| Child Stunting | 35.50 | 35.50 | Verified - No Difference |
| Child Underweight | 32.10 | 32.00 | Verified - No Difference |
| Child Wasting | 19.20 | 19.00 | Verified - No Difference |
| Child Marriage [Girl] | 23.60 | 23.30 | Verified - Negligible Difference |
| Overweight Children | 3.40 | 3.40 | Verified - No Difference |
| Clean Cooking Fuel | 55.96 | 56.20 | Verified - No Difference |
| Condom | 9.42 | 9.50 | Verified - No Difference |
| High School Matriculation [Women] | 40.96 | 41.00 | Verified - No Difference |
| Childbirths in Public Facility | 62.04 | 61.90 | Verified - No Difference |
| Currently Working Women | 24.95 | 25.40 | Verified - Negligible Difference |
| DPT Vaccination [3 Doses] | 86.70 | 86.70 | Verified - No Difference |
| Death Registration | 70.71 | 70.80 | Verified - No Difference |
| Diarrhoea Treatment [Facility] | 71.00 | 68.90 | Verified - Negligible Difference |
| Diarrhoea [Children] | 7.32 | 7.30 | Verified - No Difference |
| Diarrhoea [Received ORS] | 60.70 | 60.60 | Verified - No Difference |
| Diarrhoea [Received Zinc] | 31.50 | 30.50 | Verified - Negligible Difference |
| Early Breastfeeding Initiation | 42.20 | 41.80 | Verified - Negligible Difference |
| Elevated Blood Pressure or On Medication [Men] | 16.25 | - | Verification Not Possible - Incomparable Sample Criteria |
| Elevated Blood Pressure or On Medication [Women] | 11.69 | - | Verification Not Possible - Incomparable Sample Criteria |
| Exclusive Breastfeeding [Under 6 Months] | 63.39 | 63.40 | Verified - No Difference |
| Family Planning Services Quality [Family Planning Counselling] | 29.70 | - | Verification Not Possible - Incomparable Sample Criteria |
| Family Planning Services Quality [Side Effects Counselling] | 62.30 | 62.40 | Verified - No Difference |
| Family Planning [Any Methods by Women] | 66.69 | 66.70 | Verified - No Difference |
| Family Planning [Modern] | 56.43 | 56.50 | Verified - No Difference |
| Female Sterilization | 37.90 | 37.90 | Verified - No Difference |
| Family Planning [Unmet Need] | 9.40 | 9.40 | Verified - No Difference |
| Female School Attendance | 71.79 | 71.80 | Verified - No Difference |
| Full Vaccination | 76.90 | 77.00 | Verified - No Difference |
| Full Vaccination [Vaccination Card] | 82.60 | 83.80 | Verified - Negligible Difference |
| High Blood Sugar [Women] | 4.21 | - | Verification Not Possible - Incomparable Sample Criteria |
| Handwashing Facilities | 72.83 | 72.70 | Verified - Negligible Difference |
| Health Insurance [Any] | 40.50 | 40.30 | Verified - Negligible Difference |
| Hepatitis B Vaccine [3 Doses] | 81.90 | 83.90 | Verified - Negligible Difference |
| High Blood Sugar [Men] | 5.05 | - | Verification Not Possible - Incomparable Sample Criteria |
| High School Matriculation [Men] | 50.21 | 50.20 | Verified - Negligible Difference |
| High or Very High Blood Sugar or On Medication [Women] | 8.15 | - | Verification Not Possible - Incomparable Sample |
| High or Very High Blood Sugar or On Medication [Men] | 9.80 | - | Verification Not Possible - Incomparable Sample Criteria |
| Home Delivery by Skilled Health Personnel | 3.20 | 3.20 | Verified - No Difference |
| Hygienic Protection Methods [Menstruation] | 77.56 | 77.30 | Verified - Negligible Difference |
| ICDS Benefits [Children] | 67.60 | - | Verification Not Possible - Incomparable Sample Criteria |
| Improved Sanitation Facility | 69.95 | 70.20 | Verified - No Difference |
| Improved Source of Drinking Water | 95.87 | 95.90 | Verified - No Difference |
| ICDS Supplementary Nutrition | 69.30 | - | Verification Not Possible - Incomparable Sample Criteria |
| Institutional Childbirth | 88.60 | 89.00 | Verified - Negligible Difference |
| IUD/PPIUD | 2.10 | 2.10 | Verified - No Difference |
| Internet Usage [Women] | 33.30 | 33.30 | Verified - No Difference |
| Intimate Partner Violence [Against Women] | 29.40 | 29.30 | Verified - No Difference |
| Iodized Salt Intake | 93.90 | 93.80 | Verified - No Difference |
| Injectables | 0.60 | 0.60 | Verified - No Difference |
| Iron Folic Acid [100 days or More] | 44.30 | 44.10 | Verified - Negligible Difference |
| Iron Folic Acid [180 days or More] | 26.20 | 26.00 | Verified - No Difference |
| Literacy [Men] | 84.37 | 84.40 | Verified - No Difference |
| Literacy [Women] | 71.38 | 71.50 | Verified - No Difference |
| Low Birth Weight | 17.69 | 18.00 | Verified - Negligible Difference |
| Maternal Care Quality [Postpartum] | 89.80 | 89.50 | Verified - Negligible Difference |
| Measles-Containing Vaccine [First Dose] | 88.20 | 87.90 | Verified - Negligible Difference |
| Mild Anaemia [Women] | 24.20 | 25.60 | Verified - Negligible Difference |
| Mild Anaemia [Children] | 29.20 | 29.20 | Verified - No Difference |
| Mildly Elevated Blood Pressure [Men] | 12.04 | - | Verification Not Possible - Incomparable Sample Criteria |
| Male Sterilization | 0.30 | 0.30 | Verified - No Difference |
| Mildly Elevated Blood Pressure [Women] | 7.73 | 7.70 | Verified - No Difference |
| Moderate Anaemia [Women] | 27.80 | 28.70 | Verified - Negligible Difference |
| Moderate Anaemia [Children] | 36.50 | 35.80 | Verified - Negligible Difference |
| Measles-Containing Vaccine [Second Dose] | 59.00 | 59.00 | Verified - No Difference |
| Moderate or Severe Blood Pressure [Men] | 3.34 | - | Verification Not Possible - Incomparable Sample Criteria |
| Moderate or Severe Blood Pressure [Women] | 2.20 | - | Verification Not Possible - Incomparable Sample Criteria |
| Mother and Child Protection Card | 95.90 | 95.90 | Verified - No Difference |
| Neonatal Tetanus | 92.60 | 92.00 | Verified - Negligible Difference |
| Overweight or Obese [Women] | 23.90 | 24.00 | Verified - No Difference |
| Polio Vaccination [3 Doses] | 80.40 | 80.50 | Verified - No Difference |
| Population below 15 Years | 26.53 | 26.50 | Verified - No Difference |
| Population with BPL cards | 45.60 | 45.30 | Verified - Negligible Difference |
| Postnatal Care [Mothers] | 80.30 | 78.00 | Verified - Negligible Difference |
| Pregnancy Registration | 94.00 | 93.90 | Verified - No Difference |
| Private Latrine | 72.46 | - | Verification Not Possible - Incomparable Sample Unit |
| Probability of Dying within 28 Days | 2.47 | - | Verification Not Possible - Incomparable Estimation Units |
| Probability of Dying before Five Years | 3.74 | - | Verification Not Possible - Incomparable Estimation Units |
| Probability of Dying before One Year | 3.42 | - | Verification Not Possible - Incomparable Estimation Units |
| Receiving Solid/Semi-solid Food [6-8 Months] | 45.99 | 45.90 | Verified - No Difference |
| Risky Waist-to-hip Ratio [Women] | 58.42 | 56.70 | Verified - Negligible Difference |
| Safe Stool Disposal | 39.30 | 38.20 | Verified - Negligible Difference |
| Severe Anaemia [Women] | 2.50 | 2.40 | Verified - No Difference |
| Severe Anaemia [Children] | 2.10 | 2.10 | Verified - No Difference |
| Severe Stunting [Children] | 15.30 | 15.10 | Verified - No Difference |
| Severe Underweight [Children] | 10.30 | 10.60 | Verified - Negligible Difference |
| Severe Wasting [Children] | 7.60 | 7.70 | Verified - No Difference |
| Sexual Violence [Young Women] | 1.70 | 1.50 | Verified - No Difference |
| Skilled Birth Attendance | 89.60 | 81.40 | Verified - Negligible Difference |
| Teenage Pregnancy | 6.80 | 6.80 | Verified - No Difference |
| Tobacco Consumption [Women] | 4.10 | 4.10 | Verified - No Difference |
| Tobacco Use [Men] | 32.60 | - | Verification Not Possible - Incomparable Sample Criteria |
| Underweight [Women] | 18.70 | 18.70 | Verified - No Difference |
| Unmet Need for Spacing | 4.05 | 4.00 | Verified - No Difference |
| Very High Blood Sugar [Men] | 3.95 | - | Verification Not Possible - Incomparable Sample Criteria |
| Very High Blood Sugar [Women] | 3.31 | - | Verification Not Possible - Incomparable Sample Criteria |
| Vitamin A Dose | 67.80 | 67.50 | Verified - Negligible Difference |
| Women Participation in Household Decisions | 88.68 | 88.70 | Verified - No Difference |
| Women with Personal Mobile Phone | 54.87 | 54.00 | Verified - Negligible Difference |
| Zero Dose [Child Immunization] | 6.73 | - | Verification Not Possible - Indicator not available |
| Zero Food [Children] | 17.80 | - | Verification Not Possible - Indicator not available |
| Pill | 5.10 | 5.10 | Verified - No Difference |

*Note*. Negligible difference – 1 to 2% points; Approximation Errors in No-difference variables.

**Appendix A. Sample Code for Indicator 1: Population with Below Poverty Line Cards, 2021**

**1. Data Preparation**

- Loads the dataset

- Generate an outcome and relevant variables

**2. Model Estimation**

- IGLS (MQL1)

- MCMC (burnin(1000) chain(5000 – 25000) with cluster parexpansion)

- Extracts and save posterior betas, variances, SEs, and ESS

**3. Chain File Processing**

- Cleans and renames saved MCMC chains

- Merges all chain files and geographic key

**4. Cluster, District, and PC-level Prediction**

- Computes predicted probability for cluster, district, and PC

- Aggregates and plots cluster-, district-, and PC-level results

- Deletes temporary chain and prediction files

Below we provide an illustrative example of the data preparation, model fitting, and prediction workflow for Indicator 1: “Population with Below Poverty Line Cards” in 2021. The same modeling was applied to all indicators. The code below is written in Stata and uses the runmlwin command for multilevel logistic regression with both IGLS and MCMC estimation.

**NFHS 5 Population with BPL Cards (IPI Indicator Number – 1)**

*** IPI Estimation**

*** (1) PREPARE INDIVIDUAL-LEVEL DATA AND CREATE OUTCOME**

*** Load the file // Please adjust the path based on your file location**

use "IAPR7DFL_district722.dta", clear

*** Keep required variables**

keep hhid hvidx hv000 hv001 hv002 hv005 hv006 hv007 hv008 hv009 hv011 hv021 hv024 hv025 shdist sh75 newstate dist_code dist_name

*** Sample Weights Construction**

gen wt = hv005/1000000

*** Sample exclusion - Indicator-specific criteria**

drop if sh75==8 // dropped missing observation - 4,642

*** Final analytic sample - 2,839,275 sample population**

*** Indicator construction - bplcard**

gen bplcard05 = .

replace bplcard05=1 if sh75==1

replace bplcard05=0 if sh75==0

label define bplcard05 1 "Yes" 0 "No"

label values bplcard05 bplcard05

*** Quality check - verification - India NFHS5 report - Page 45, Table 2.11, Prevalence 45.3%**

tabstat bplcard05 [aw=wt]

tab bplcard05 [aw=wt] // Actual Estimate - 45.5%

drop if missing(bplcard05)

*** Rename variable to indicator number**

rename bplcard05 ind05_01

*** Generate variables for MLwiN**

rename hv024 state

rename shdist district

rename hv001 cluster

local N_start = _N

gen cons = 1

gen case = _n

save "data_ind05_01", replace

*** (2) Run MLwiN model and save parameter summary**

global MLwiN_path "mlnscript" // Please adjust the path based on your file location

sort state district cluster

*** Step 1: IGLS initialization (MQL1) (to provide starting values for MCMC)**

*** Fit model by IGLS (MQL1) to initialize**

xi: runmlwin ind05_01 cons, ///

level4(state: cons) ///

level3(district: cons) ///

level2(cluster: cons) ///

level1(case:) ///

discrete(distribution(binomial) link(logit) denominator(cons)) ///

mlwinsettings(optimat) nopause

*** Step 2: MCMC (option: burnin(1000) chain(5000 - 25000) thinning(10), with cluster parexpansion)**

*** Fit model by MCMC**

runmlwin ind05_01 cons, ///

level4(state: cons, residuals(f, savechains("state_chains_ind05_01.dta", replace))) ///

level3(district: cons, residuals(v, savechains("district_chains_ind05_01.dta", replace))) ///

level2(cluster: cons, parexpansion residuals(u, savechains("cluster_chains_ind05_01.dta", replace))) ///

level1(case:) ///

discrete(distribution(binomial) link(logit) denominator(cons)) ///

mcmc(burnin(500) chain(15000) thinning(10) orthogonal hcentring(4) ///

savechains("parameter_chains_ind05_01.dta", replace)) ///

initsprevious ///

nopause

*** Extract posterior summaries and save** //SE values may differ slightly

clear

set obs 1

gen str20 outcome = "ind05_1"

gen beta0 = e(b)[1,1]

gen beta0_se = e(sd)[1,1]

gen f_var = e(b)[1,2]

gen f_var_se = e(sd)[1,2]

gen f_var_ess = e(ess)[1,2]

gen v_var = e(b)[1,3]

gen v_var_se = e(sd)[1,3]

gen v_var_ess = e(ess)[1,3]

gen u_var = e(b)[1,4]

gen u_var_se = e(sd)[1,4]

gen u_var_ess = e(ess)[1,4]

*** Save final summary**

save "summary_ind05_1.dta", replace

*** (3) PREPARE AND MERGE MCMC CHAINS FOR PREDICTION**

*** Step 1: Prepare all chain files**

use "parameter_chains_ind05_01.dta", clear

keep iteration FP1_cons

rename FP1_cons beta0

compress

save "parameter_chains_ind05_01.dta", replace

use "state_chains_ind05_01.dta", clear

keep iteration state value

rename value f

compress

save "state_chains_ind05_01.dta", replace

use "district_chains_ind05_01.dta", clear

keep iteration district value

rename value v

compress

save "district_chains_ind05_01.dta", replace

use "cluster_chains_ind05_01.dta", clear

keep iteration cluster value

rename value u

compress

save "cluster_chains_ind05_01.dta", replace

*** Step 2: Create cluster→district→state key**

use "data_ind05_01", clear

keep cluster state district newstate dist_code

duplicates drop

isid cluster

*** Step 3: Merge all chains into prediction dataset**

cross using "parameter_chains_ind05_01.dta"

duplicates drop

isid cluster iteration

merge m:1 state iteration using "state_chains_ind05_01.dta", assert(match) nogenerate

merge m:1 district iteration using "district_chains_ind05_01.dta", assert(match) nogenerate

merge m:1 cluster iteration using "cluster_chains_ind05_01.dta", assert(match) nogenerate

compress

save "predictions_ind05_01.dta", replace

*** Step 4: Cluster-level predictions // Please use updated ID**

use "predictions_ind05_01.dta", clear

gen p = invlogit(beta0 + f + v + u)

gcollapse (mean) p_mean = p (sd) p_sd = p (p2.5) p_ll = p (p97.5) p_ul = p, by(newstate dist_code cluster)

replace p_mean = p_mean * 100

replace p_sd = p_sd * 100

replace p_ll = p_ll * 100

replace p_ul = p_ul * 100

sort p_mean

gen p_mean_rank = _n

twoway (rspike p_ul p_ll p_mean_rank) (scatter p_mean p_mean_rank), ///

ytitle("Cluster probability") xtitle("Rank") legend(off) ///

name("Figure1", replace)

graph export "Figure1_ind05_01_cluster.png", name("Figure1", replace) width(1000) replace

rename p_mean p_mean_ind05_01

rename p_sd p_sd_ind05_01

rename p_ll p_ll_ind05_01

rename p_ul p_ul_ind05_01

rename p_mean_rank p_mean_rank_ind05_01

save "cluster_estimates_ind05_01.dta", replace

*** Step 5: District-level predictions // Please use updated District ID**

use "predictions_ind05_01.dta", clear

gen p = invlogit(beta0 + f + v + u)

collapse (mean) q = p (max) newstate, by(dist_code iteration)

gcollapse (mean) q_mean = q (sd) q_sd = q (p2.5) q_ll = q (p97.5) q_ul = q, by(newstate dist_code)

replace q_mean = q_mean * 100

replace q_sd = q_sd * 100

replace q_ll = q_ll * 100

replace q_ul = q_ul * 100

sort q_mean

gen q_mean_rank = _n

twoway (rspike q_ul q_ll q_mean_rank) (scatter q_mean q_mean_rank), ///

ytitle("District mean of the cluster probabilities") xtitle("Rank") legend(off) ///

name("Figure2", replace)

graph export "Figure2_ind05_01_district.png", name("Figure2", replace) width(1000) replace

rename q_mean q_mean_ind05_01

rename q_sd q_sd_ind05_01

rename q_ll q_ll_ind05_01

rename q_ul q_ul_ind05_01

rename q_mean_rank q_mean_rank_ind05_01

save “district_estimates_ind05_01.dta", replace

*** Step 6: PC-level predictions // Please use updated PC ID**

*** Step 6.1 Merge Cluster Predictions with Cluster-to-PC linkage files**

use "predictions_ind05_01.dta", clear

sort cluster

save, replace

use "NFHS-5_Final_File.dta", clear

sort cluster

save, replace

use "predictions_ind05_01.dta", clear

merge m:1 cluster using "NFHS-5_Final_File.dta"

keep if _merge==3

*** Step 6.2: Collapse for PC level Estimates using PC-ID**

gen p = invlogit(beta0 + f + v + u)

collapse (mean) q = p (max) newstate, by(pc_idnew iteration)

gcollapse (mean) q_mean = q (sd) q_sd = q (p2.5) q_ll = q (p97.5) q_ul = q, by(newstate pc_idnew)

replace q_mean = q_mean * 100

replace q_sd = q_sd * 100

replace q_ll = q_ll * 100

replace q_ul = q_ul * 100

sort q_mean

gen q_mean_rank = _n

twoway (rspike q_ul q_ll q_mean_rank) (scatter q_mean q_mean_rank), ///

ytitle("PC mean of the cluster probabilities") xtitle("Rank") legend(off) ///

name("Figure3", replace)

graph export "Figure3_ind05_01_pc.png", name("Figure3", replace) width(1000) replace

rename q_mean q_mean_ind05_01

rename q_sd q_sd_ind05_01

rename q_ll q_ll_ind05_01

rename q_ul q_ul_ind05_01

rename q_mean_rank q_mean_rank_ind05_01

save “pc_estimates_ind05_01.dta", replace

***Step 7: Clean up temporary files**

erase "state_chains_ind05_1.dta"

erase "district_chains_ind05_1.dta"

erase "cluster_chains_ind05_1.dta"

erase "parameter_chains_ind05_1.dta"

erase "predictions_ind05_1.dta"
